# Artificial Light‐Harvesting Complexes Enable Rieske Oxygenase Catalyzed Hydroxylations in Non‐Photosynthetic cells

**DOI:** 10.1002/anie.201914519

**Published:** 2020-01-24

**Authors:** F. Feyza Özgen, Michael E. Runda, Bastien O. Burek, Peter Wied, Jonathan Z. Bloh, Robert Kourist, Sandy Schmidt

**Affiliations:** ^1^ Institute of Molecular Biotechnology Graz University of Technology Petersgasse 14/1 8010 Graz Austria; ^2^ DECHEMA-Forschungsinstitut Theodor-Heuss-Allee 25 60486 Frankfurt am Main Germany

**Keywords:** biocatalysis, oxyfunctionalization, photocatalysis, photoinduced electron transfer, Rieske dioxygenases

## Abstract

In this study, we coupled a well‐established whole‐cell system based on E. coli via light‐harvesting complexes to Rieske oxygenase (RO)‐catalyzed hydroxylations in vivo. Although these enzymes represent very promising biocatalysts, their practical applicability is hampered by their dependency on NAD(P)H as well as their multicomponent nature and intrinsic instability in cell‐free systems. In order to explore the boundaries of E. coli as chassis for artificial photosynthesis, and due to the reported instability of ROs, we used these challenging enzymes as a model system. The light‐driven approach relies on light‐harvesting complexes such as eosin Y, 5(6)‐carboxyeosin, and rose bengal and sacrificial electron donors (EDTA, MOPS, and MES) that were easily taken up by the cells. The obtained product formations of up to 1.3 g L^−1^ and rates of up to 1.6 mm h^−1^ demonstrate that this is a comparable approach to typical whole‐cell transformations in E. coli. The applicability of this photocatalytic synthesis has been demonstrated and represents the first example of a photoinduced RO system.

## Graphical abstract

**Illuminate me**! The photoactivation of Rieske dioxygenases in the absence of glucose or any cofactor was successfully conducted using several photosensitizers for the bioconversion of three different substrates, which represents the first example of a photoinduced Rieske system.

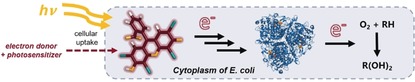

Nature's creativity in developing solutions for functionalization reactions like hydroxylations at activated or non‐activated C−H bonds is remarkably shown by an expansive list of metal‐dependent enzymes.^1^, ^2^ These enzymes, like the Rieske non‐heme iron oxygenases (ROs), are able to activate molecular oxygen in order to generate reactive oxygen species capable of hydroxylating alkyl substrates, but also to promote further oxidative transformations.^3^, ^4^, ^5^, ^6^, ^7^, ^8^, ^9^, ^10^, ^11^ For many of these reactions no “classical” chemical counterpart is known. Due to their dependency on complex electron transport chains^12^ as well as the requirement of an efficient in situ cofactor regeneration, the majority of synthetic applications of ROs relies on recombinant whole‐cell catalysts. Generally, for such reactions various concepts have been developed that rely on electron supply via the metabolism of living heterotrophic cells.^13^, ^14^, ^15^ In synthetic applications, the nicotinamide cofactors are recycled by using energy‐rich organic molecules as electron donors. In most cases, only a small fraction of the electrons provided by these sacrificial co‐substrates is utilized, resulting in a poor atom efficiency.^14^ Moreover, when glucose is supplied as the sacrificial substrate for the recycling of NADPH, the often‐used glucose dehydrogenase utilizes only a portion of the electron pairs from each glucose molecule. In order to solve this challenge, many alternative solutions are currently under consideration.^16^, ^17^, ^18^ Next to linking photochemistry to enzymes in vitro for cofactor regeneration,^18^, ^19^, ^20^, ^21^, ^22^, ^23^, ^24^ autotrophic and chemolithoautotrophic organisms have recently received attention as they are capable of utilizing inorganic compounds as electron donors.^25^, ^26^, ^27^, ^28^, ^29^ Light‐driven whole‐cell reactions in cyanobacteria show the same reaction rates as *E. coli*.^26^, ^27^, ^29^ Yet, the strong absorption of the photosynthetic apparatus lead to self‐shading of the cells at high densities, thus resulting in a low light utilization and a reduced photosynthetic activity.^30^ On the other hand, introducing artificial photosynthesis in heterotrophic bacteria such as *E. coli* offers the advantage of utilizing a genetically easy‐to‐manipulate organism along with the capability of producing high amounts of soluble protein within the cells. Additionally, these systems are less prone to the inhibiting effects of self‐shading at high cell densities. Currently reported artificial light‐driven approaches in heterotrophs comprise the use of inorganic–biological hybrid systems and the coupling of organic photosensitizers to biotransformations in vivo.^31^, ^32^ One of the earliest examples of a whole‐cell reaction was reported using recombinant *E. coli* coupled to photocatalytic H_2_ production via an extracellular photosensitizer (TiO_2_) and methyl viologen as the electron mediator.^31^ Similarly, the light‐driven H_2_ evolution and C=C or C=O bond hydrogenation by *Shewanella oneidensis* using methyl viologen was shown.^33^ These are interesting systems, however, the toxicity of methyl viologen is well known, thus hampering large‐scale applications. A direct and perhaps the most applicable approach has been reported by Park and co‐workers.^32^ This light‐driven catalysis is based on in vivo photoreduction of a P450 by using different fluorescent dyes and sacrificial electron donors.^32^ Although operating at low product concentrations, it represents a highly promising system for the challenging multicomponent ROs since these enzymes usually exhibit high catalytic activities despite low expression levels, and thus a high potential for artificial photosynthesis approaches in *E. coli* (Figure 1).

**Figure 1:**
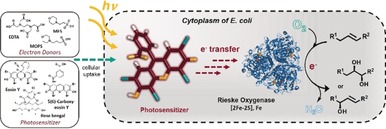
In vivo photoactivation of a Rieske non‐heme iron oxygenase by an artificial light‐harvesting complex. The catalytic turnover of the oxygenase component is mediated by the excited photosensitizer that transfers electrons from the sacrificial electron donor to the oxygenase within the cytoplasm of *E. coli*.

Herein, we demonstrate the general feasibility of this light‐induced approach together with a characterization of the crucial parameters determining the catalytic efficiencies of the light‐driven in vivo enzymatic reaction.

We explored eosin Y (2,4,5,7‐tetrabromofluorescein, EY) and its xanthene derivatives as well as safranin O (SO) as efficient photosensitizers to drive RO‐catalyzed hydroxylation reactions under illumination with LED or fluorescent lamps. As electron donors, either 3‐(*N*‐morpholino)propanesulfonic acid (MOPS), 2‐(*N*‐morpholino)ethanesulfonic acid (MES), and ethylenediaminetetraacetic acid (EDTA) were investigated.^23^ In this way, the successive transfer of electrons reduces the catalytic iron and drives the conversion of (*R*)‐limonene (**1**) into (1*R*,5*S*)‐carveol (**1 a**), toluene (**2**) into benzyl alcohol (**2 a**), and indene (**3**) to 1‐indenol (**3 a**) and *cis*‐ or *trans*‐1,2‐indanediol (**3 b**) by the ROs under visible‐light irradiation and without the need of NAD(P)H as redox partner (Scheme 1).

**Scheme 1:**
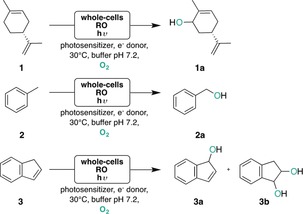
Light‐driven whole‐cell oxyfunctionalization reactions catalyzed by CDO or NDO.

We chose cumene dioxygenase (CDO, from *Pseudomonas fluorescens* IP01) and naphthalene dioxygenase (NDO, from *Pseudomonas* sp. NCIB 9816‐4) as model enzymes (Figure S1, Tables S1–S7) since both enzymes have been extensively studied for a long time and many redesigned variants have been investigated. We became particularly interested in two variants of CDO and NDO that have been engineered toward the asymmetric dihydroxylation of olefins. CDO variant M232A converts **1** almost exclusively to **1 a** (*ee* >98 %),^34^ whereas NDO variant H295A shows a different ratio between allylic monohydroxylation and *cis*‐dihydroxylation for several substituted arenes.^35^ First, we investigated the expression of the whole RO system under different culture conditions (SDS‐PAGE, Figure S2) and confirmed the RO activity by an agar plate assay based on indigo formation (Figures S3 and S4) and could identify significant activity toward indole.^36^

To identify the cell density leading to the highest product formation catalyzed by CDO expressed under different culture conditions, biotransformations supplemented with glucose (20 mm) and with **1** (10 mm) as substrate were performed under dark conditions (Table S8). As expected, CDO‐containing whole cells (100 g_WCW_ L^−1^) expressed in TB medium at 30 °C gave the highest activity for **1** and the product **1 a** was obtained with an *ee* of >99 % (Figure S5). The obtained product concentrations of **1 a** and **2 a** were lower than previously reported,^34^ which we mostly attribute to a different expression protocol (19 hours instead of 2 hours) and a lower cell density (100 g_WCW_ L^−1^ instead of 200 g_WCW_ L^−1^) than previously reported.^34^ However, we first investigated the light‐driven system by using CDO‐containing whole cells at 100 g_WCW_ L^−1^ in order to avoid self‐shading at too high cell densities and used cells expressed under the conditions mentioned above (19 hours).

We became interested in different photosensitizer/electron donor combinations to drive the light‐driven whole‐cell hydroxylation catalyzed by the ROs (Figures S6–S12, Table 1, Table S9 and 10). We first chose MES since it has been successfully used as efficient electron donor previously,^37^ is nontoxic, and can be up taken by *E. coli* cells.^38^, ^39^ The *E. coli* strain used herein is lacking a natural uptake system for flavins,^40^ thus we decided to choose a PS that can easily enter the cells^32^ while showing similar redox properties to flavins. 5(6)‐Carboxyeosin (CE) was chosen first since it possesses excellent photosensitizer properties (Figure S8) with an *E*_Redox_ of −1.06 V, which is similar to the *E*_Redox_ of proflavine.^22^ Performing the photoenzymatic hydroxylation of **1** and **2** with 50 mm MES and 100 μm 5(6)‐carboxyeosin (CE) at a cell density of 100 g_WCW_ L^−1^ resulted in a smooth formation of the desired products **1 a** (up to ≈360 μm in 24 hours) and **2 a** (up to ≈200 μm in 24 hours) under illumination with white light. Nonetheless, the obtained concentrations of **1 a** and **2 a** always remained low (≤360 μm, Table 1). Due to the known toxicity of **1** as well as **1 a** on whole cells, we turned our attention toward indene **3** as a typical substrate for ROs. In the next set of experiments, we investigated the same photosensitizer/electron donor combination for both model enzymes (Table 1) and were pleased to find that conversions of up to 85 % were achieved with **3**. Since **3** was the most promising substrate for the in‐depth characterization of our light‐driven system, we turned our attention to the investigation of further photosensitizer/electron donor combinations. We investigated EY, rose bengal (RB), and CE with EDTA, as well as with MOPS and MES as sacrificial electron donors that can be up taken by cells (Table 2).^22^, ^39^, ^41^, ^42^ The electron donor can constitute an obstacle in this photobiocatalytic setup,^23^ as EDTA may suffer, for example, from incompatibility with the RO due to its ability to sequester the Fe^3+^ ion located in the active site of the oxygenase. However, we did not see any activity loss of NDO H295A and CDO M232A in dark reactions supplemented with EDTA (Figure S13) nor any toxicity effects of MES/MOPS on the cells (Figure S14). Moreover, when we performed the light‐driven hydroxylations with lysed cells, product concentrations of only 5.6 mm compared to 8.5 mm with whole cells were achieved (Table S11), indicating that the cells do not suffer from the electron donors or their decomposition products. The obtained product conversions with **3** as substrate are summarized in Table 2. Reactions supplemented with EDTA led in general to a lower product formation than reactions with MOPS and MES. However, also with EDTA product concentrations of up to 7.5 mm could be achieved, leading to the assumption that EDTA is not sequestering the catalytic iron ion from the enzyme's active site. Moreover, we were pleased to find that the utilization of **3** as substrate boosted the product formation to the mm range, leading to a conversion of up to >85 % within 24 hours when CE was used in combination with MES (Tables 1 and 2). The determination of the incident photon flux density (Figure S11 B) revealed that the light intensity at each position of the light reactor varies in a range between 32–112 μE L^−1^ s^−1^, which causes a light‐intensity‐dependent photochemical background reaction. This photochemical background reaction is only observed when **3** is used as substrate and leads to the accumulation of *trans*‐**3 b** within 6–20 hours of reaction (Figures S15–S17). Moreover, 1‐indanone formation was observed, which we attribute to an isomerization reaction of **3 a** (Table S12).

**Table 1:** Photobiocatalytic hydroxylation of (*R*)‐limonene **1**, toluene **2** and indene **3** catalyzed by CDO M232A and NDO H295A, respectively, under dark and light conditions.

Enzyme	Reaction	Substrate	Product		Whole‐cell
	conditions		conc. [mm]^[a]^	*de* or *dr* [%]^[b]^	activity^[c]^ [mU g_WCW_^−1^]
CDO M232A	dark/glucose	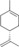	1.1±0.1	>99	8.0
	dark/CE/MES		0.1±0.04		n.b.
	light/CE/MES		0.4±0.05		2.5
empty vector	light/CE/MES	**1**	0	n.d.	n.d.
NDO H295A	dark/glucose	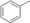	0.6±0.02	n.a.	8.8
	dark/CE/MES		0		n.d.
	light/CE/MES		0.2±0.01		2.8
empty vector	light/CE/MES	**2**	0		n.d.
CDO M232A	dark/glucose	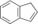	4.8±0.8	100:0	n.d.
	dark/CE/MES		1.5±0.2	100:0	n.d.
	light/CE/MES		8.3±0.08	90:10	124
NDO H295A	dark/glucose		2.3±0.17	100:0	n.d.
	dark/CE/MES		0.5±0.03	n.a.	n.d.
	light/CE/MES		8.5±0.4	86:14	107
empty vector	light/CE/MES	**3**	0.7±0.2	18:82	n.d.

Reaction conditions dark: [substrate]=10 mm, [glucose]=20 mm, [whole cells]=100 g_WCW_ L^−1^ (*E. coli* JM109 (DE3)_pDTG141_NDO H295A or *E. coli* JM109_pCDOv1_CDO M232A), sodium phosphate buffer (pH 7.2, 50 mm), 24 hours. Reaction conditions light: [substrate]=10 mm, [whole cells]=100 g_WCW_ L^−1^ (*E. coli* JM109 (DE3)_pDTG141_NDO H295A or *E. coli* JM109_pCDOv1_CDO M232A), MES buffer (50 mm), white light illumination (max. 112 μE L^−1^ s^−1^) 24 hours; n.a. not applicable; n.d. not determined. [a] For **3**, product concentrations refer to the sum of **3 a** and **3 b**. [b] Diastereomeric ratio *cis:trans*‐**3 b** was determined after 4–6 hours of reaction. [c] Determined from the linear range of product formation determined from the kinetic profiles for each reaction (Figures S20–S25).

**Table 2:** The combination of photosensitizer and electron donor is a crucial factor for the efficiency of the light‐driven reaction. 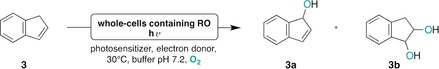

Enzyme	Photo‐ sensitizer/		Products				Whole‐cell activity			Apparent quantum yield
	Electron donor		Max. conc. [mm]^[a]^	Diast. ratio *cis/trans*‐**3 b** [%]^[b]^	Distrib. **3 a**/**3 b** [%]^[c]^		Specific activity^[d]^ [mU g_WCW_^−1^]	Productivity [mm h^−1^]		[%]
CDO M232A	EY/EDTA		3.7±0.4	100:0	3:97		29	0.18		0.09
	EY/MOPS		6.8±0.03	100:0	0:100		21	0.13		0.06
	EY/MES		4.7±0.2	100:0	4:96		41	0.25		0.12
	RB/EDTA		6.8±0.3	96:4	4:96		265	1.59		0.78
	RB/MOPS		7.3±0.6	95:5	4:96		156	0.94		0.46
	RB/MES		5.8±0.4	95:5	3:97		275	1.65		0.81
	CE/EDTA		4.0±0.01	94:6	2:98		43	0.26		0.13
	CE/MOPS		8.6±0.6	88:12	0:100		102	0.61		0.30
	CE/MES		8.3±0.08	90:10	0:100		124	0.75		0.37
NDO H295A	EY/EDTA		4.7±0.2	96:4	4:96		47	0.23		0.11
	EY/MOPS		7.7±0.4	83:17	2:98		61	0.37		0.18
	EY/MES		7.4±0.3	86:14	4:96		87	0.52		0.26
	RB/EDTA		7.5±1.0	97:3	0:100		53	0.32		0.16
	RB/MOPS		7.5±1.2	95:5	2:98		148	0.89		0.44
	RB/MES		7.9±0.6	97:3	3:97		79	0.45		0.22
	CE/EDTA		5.5±0.5	94:6	6:94		50	0.30		0.15
	CE/MOPS		7.3±0.4	95:5	18:82		143	0.86		0.42
	CE/MES		8.5±0.4	86:14	0:100		107	0.64		0.32

Reaction conditions: [**3**]=10 mm; [whole cells]=100 g_WCW_ L^−1^ (*E. coli* JM109 (DE3)_pDTG141_NDO H295A or *E. coli* JM109_pCDOv1_CDO M232A); sodium phosphate buffer (pH 7.2, 50 mm) when using 25 mm EDTA, otherwise 50 mm MES/MOPS buffer; white‐light illumination (max. 112 μE L^−1^ s^−1^).[a] Sum of **3 a** and **3 b**; time points for determination were chosen at maximum product concentration during the time course of the reaction. [b] The diastereomeric ratio was determined after 4–6 hours of reaction.[c] Determined after 24 hours.[d] Determined from the liner range of product formation determined from the kinetic profiles for each reaction (Figures S19–S24).

To determine the incident photon flux, chemical actinometry was performed using the well‐described ferrioxalate actinometer (Table 2 and Table S10).^42^ Although the cell suspension showed strong scattering and optical absorption by other cell or solution components, we were able to estimate quantum yields (QY, Table S10). Additionally, apparent quantum yields (AQY) were calculated as the ratio of two times the observed product formation rate to the incident photon flux, as two photons are required per turnover (Table 2). Admittedly, the given AQYs are lower than the typical values achieved in photochemical reactions; however, these values lay a promising foundation for further optimization of this artificial photosynthetic systems.

To investigate whether the observed product formation was strictly light‐dependent and only proceeded through the electron transfer mediated by the photosensitizer, we conducted control reactions with an empty vector control, but performed in light and dark with and without an electron donor (Figure 2 A, Figure S19). Indeed, the resulting product formations with the empty vector controls were much lower under dark and light conditions. We attribute the turnover in the dark to the production of carbohydrates, which under “starvation conditions” were consumed to regenerate NAD(P)H.^26^, ^27^, ^43^, ^44^ However, under light conditions a photochemical background reaction was observed, which varies depending on the applied light intensity between 0.15 to 3.5 mm and depending on the applied photosensitizer/electron donor combination (Figure 2 A, Figure S19). The background reaction contributed up to 12 % of total product formation when CE/MOPS was used and 35 % when RB/MOPS was used (Figure 2 A), indicating that the photochemical background reaction depends on the photosensitizer. This capability seems to be limited by the applied light intensity, since the overall background reaction remained low in all cases (<0.5 mm) when lower light intensities were used, thus confirming that the reaction is truly light‐driven.

**Figure 2:**
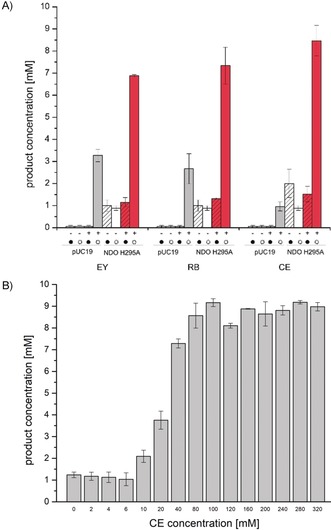
A) Control reactions using NDO H295A under light (○) and dark (•) conditions with (+) and without (−) 100 μm EY, RB, or CE in the presence of NDO H295A (red bars) or with an empty vector control (gray bars) in 50 mm MOPS. Values for the empty vector control were the highest that have been achieved when the maximum light intensity of 112 μE L^−1^ s^−1^ was applied. B) Effect of photosensitizer concentration on product yield. Different concentrations of CE used in combination with MES as electron donor in the light‐driven whole‐cell hydroxylation reaction employing NDO H295A. Reaction conditions: 0–320 μm photosensitizer, 10 mm **3**, 50 mm MES, 100 g_WCW_ L^−1^ whole cells (*E. coli* JM109 (DE3)_pDTG141_NDO H295A, 19 h expression), 50 mm MES, white light (max. 112 μE L^−1^ s^−1^), 30 °C, 140 rpm.

Additionally, we investigated the influence of the photosensitizer (Figure 2 B) and electron donor concentration, as well as the cell density (Figure S18) on the efficiency of the photocatalytic activation of NDO H295A. Under light illumination, the RO‐catalyzed hydroxylation of **3** was most efficient when CE concentrations of 80 to 100 μm were used. Between 10 μm and 80 μm of CE, an increase in product formation was observed (Figure 2 B). However, when >100 μm of CE was used, no further increase was seen; that is, above 100 μm CE either the concentration is not limited or the transport of the photosensitizer inside the cells is hampered (Figure 2 B). However, we observed photobleaching over time. When an additional 100 μm of CE was added to the light‐driven hydroxylation, the product formation accelerated again, and 6 hours after the addition of further CE already 7.2 mm of product had formed, in contrast to only 3 mm without the addition of more CE (Figure 3 A).

**Figure 3:**
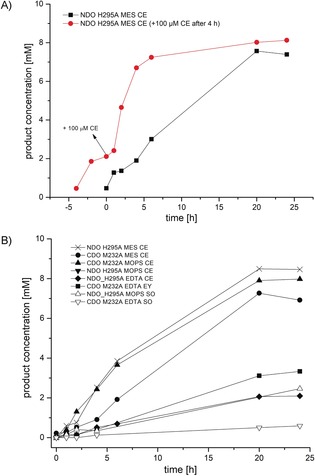
A) Effect of photobleaching of CE on the time course of the light‐driven hydroxylation catalyzed by NDO H295A. The red curve visualizes the addition of further 100 μm CE after 4 h of biotransformation, whereas the black curve shows the light‐driven biotransformation without additional CE. B) Kinetic profile obtained for the light‐driven whole‐cell hydroxylation reaction employing CDO M232A and NDO H295A with SO, CE, and EY in combination with either EDTA, MOPS, or MES as electron donors. Reaction conditions: 100 μm photosensitizer, 10 mm **3**, 25 mm EDTA or 50 mm MOPS/MES, in A) 25–300 g_WCW_ L^−1^ and in B) 100 g_WCW_ L^−1^ whole cells (*E. coli* JM109 (DE3)_pDTG141_NDO H295A or *E. coli* JM109_pCDOv1_CDO M232A, 19 h expression), white light (max. 112 μE L^−1^ s^−1^), 30 °C, 140 rpm, 24 hours.

We further investigated the effect of increasing cell density on the efficiency of the light‐driven hydroxylation reaction (Figure S18). When the cell density as well as the electron donor concentration (CE constant) is increased, it can be seen that the product formation is influenced by the concentration of the electron donor when the cell density is higher, because more photoinduced electrons are transferred to the enzyme. However, the system seems to be limited by the applied light intensities (max. 112 μE L^−1^ s^−1^); that is, from a certain cell density on, the product concentration is not “controlled” anymore by the concentration of the electron donor, and at that point the light intensity in the system becomes the limiting factor.

Light intensity plays a crucial role on the efficiency of the light‐driven catalysis and influences the extent of the photochemical background reaction. When the light intensity was reduced by 75 % (Figure S10 B), the obtained product concentrations decreased by only 20 %.

Finally, we followed the light‐driven reaction in a time‐course experiment over 24 hours under optimized conditions (Figure 3 B). The product formation proceeded smoothly within 24 hours of reaction; however, in the case of NDO H295A/MES/CE and CDO M232A/MOPS/CE no significant product increase was observed after 20 hours. Noteworthy, when SO was used in combination with MOPS or EDTA, the obtained product concentrations remained always lower than with other photosensitizer/electron donor combinations.

To conclude, we have shown the photoactivation of two different ROs in an *E. coli* based whole‐cell system by coupling light‐harvesting complexes to hydroxylation reactions in vivo. This was successfully demonstrated by using several photosensitizers for the bioconversion of three different substrates, hence representing the first example of photoinduced RO systems. Particularly for challenging multicomponent oxygenases, this system offers the advantage of relying on the well‐studied genetic toolbox of *E. coli* as the host, thereby facilitating a broad applicability of light‐driven artificial photosynthesis. The obtained product formations of up to 1.3 g L^−1^ and rates of up to 1.6 mm h^−1^ demonstrate that competitive productivities comparable to those of cyanobacteria were achieved.^28^

The coupling of artificial light‐harvesting complexes to enzymes inside cells provides a versatile route to accessing diverse and selective visible‐light‐driven chemical syntheses especially when unstable or multicomponent enzymes are used.

## Conflict of interest

The authors declare no conflict of interest.

## Supporting information

As a service to our authors and readers, this journal provides supporting information supplied by the authors. Such materials are peer reviewed and may be re‐organized for online delivery, but are not copy‐edited or typeset. Technical support issues arising from supporting information (other than missing files) should be addressed to the authors.

Supplementary
