# Supplementary material for: Artificial Light‐Harvesting Complexes Enable Rieske Oxygenase Catalyzed Hydroxylations in Non‐Photosynthetic cells
Source: Angew Chem Int Ed Engl. 2020 Jan 24;59(10):3982–7. doi: 10.1002/anie.201914519 (PMC7065155; doi:10.1002/anie.201914519)
Supplement: Supplementary file 1 — Supplementary [file ANIE-59-3982-s001.pdf]

Angewandte Chemie
Eine Zeitschrift der Gesellschaft Deutscher Chemiker

Supporting Information

Artificial Light-Harvesting Complexes Enable Rieske Oxygenase Catalyzed Hydroxylations in Non-Photosynthetic cells

*F. Feyza Özgen, Michael E. Runda, Bastien O. Burek, Peter Wied, Jonathan Z. Bloh, Robert Kourist, and Sandy Schmidt**

anie_201914519_sm_miscellaneous_information.pdf

LIST OF ABBREVIATIONS

**AQY**	apparent quantum yield
**CE**	5(6)-carboxyeosin Y
**CDO**	Cumene dioxygenase
**WCW**	wet cell weight
**DCM**	dichloromethane
de	diastereomeric excess
**EDTA**	ethylenediaminetetraacetic acid
ee	enantiomeric excess
**EY**	Eosin Y
**FAD**	flavin adenine dinucleotide
**FMN**	flavin mononucleotide
**GC-FID**	gas chromatography – flame ionization
**GC-MS**	gas chromatography – mass spectrometry
**H_2O_2**	hydrogen peroxide
**LB**	*lysogeny* broth
**LED**	light emitting diode
**MES**	2-(N-morpholino)ethanesulfonic acid
**MOPS**	3-(N-morpholino)propanesulfonic acid
**NAD(P)H**	nicotinamide adenine dinucleotide (phosphate)
**NDO**	Naphtalene dioxygenase
**nt**	nucleotide
**O_2**	molecular oxygen
**ONC**	over-night culture
**PCR**	polymerase chain reaction
**PLA**	polylactide
**QY**	true quantum yield
**RB**	rose bengal
**ROs**	Rieske non-heme iron oxygenases
**SO**	safranin O
**SDS-PAGE**	sodium dodecyl sulfate - polyacrylamide gel electrophoresis
**SOC**	super optimal broth with catabolite repression
**SPB**	sodium phosphate buffer
**TB**	terrific broth
**Tm**	melting temperature
**WT**	wild-type

EXPERIMENTAL PROCEDURES

GENERAL

Unless stated otherwise all chemicals, solvents and buffers were purchased from Sigma-Aldrich (Steinheim, Germany) or Carl-Roth GmbH (Karlsruhe, Germany) in the highest purity available and used without further purification. Restriction enzymes and polymerases were used from Thermo Scientific (Schwerte, Germany), PfuPlus polymerase from Promega (Mannheim, Germany) and enzymes for Gibson Assembly from NEB (Frankfurt am Main, Germany). Kits for PCR product purification, gel extraction of DNA and plasmid isolations were obtained from Thermo Scientific (Schwerte, Germany). DNA Sequencing was performed by GATC Biotech GmbH (Konstanz, Germany) or Microsynth AG (Balgach, Switzerland). GC-MS analyses were performed on a Shimadzu GCMS-QP2010 system equipped with an AOC-20i auto injector. GC-FID analyses were performed on a Shimadzu GC-2010 or Nexis GC-2030 system equipped with an AOC-20i auto injector. Columns and conditions for GC analyses are indicated separately.

BACTERIAL STRAINS, PLASMIDS AND PRIMERS

For routine cloning and expression, chemically competent *E. coli* strains were used. All strains used for cloning or protein expression are listed below (Table S1).

Table S1: Strains used for molecular cloning and protein expression.

Strain	Genotype	Description/Use	Source
**JM109**	*endA1, recA1, gyrA96, thi, hsdR17 (rk–, mk+), relA1, supE44, Δ(lac-proAB), [F′ traD36, proAB, laqIqZΔM15]*	routine cloning and expression	New England Biolabs, Inc.
**JM109 (DE3)**	*endA1, recA1, gyrA96, thi, hsdR17 (rk–, mk+), relA1, supE44, λ–, Δ(lac-proAB), [F′, traD36, proAB, lacIqZΔM15], lDE3*	routine cloning and expression of T7-based vectors	Promega Corp.
**TOP10**	*F- mcrA Δ(mrr-hsdRMS-mcrBC) φ80(lacZ)ΔM15 Δ lacX74 recA1 araD139 Δ(araleu)7697 galU galK rpsL (StrR) endA1 nupG*	routine cloning and expression	Thermo Fisher Scientific
**Mach1**	*F- φ80(lacZ)ΔM15 ΔlacX74 hsdR(rK-mK+) ΔrecA1398 endA1 tonA*	routine cloning, fast growth (dt=50 min)	Thermo Fisher Scientific

The plasmid pDTG141 carrying the nah genes encoding for NDO oxygenase α- (NahAc) and β- (NahAd) subunits, ferredoxin (NahAb) and reductase (NahAa) from *Pseudomonas* sp. strain NCIB 9816-4 was obtained from Rebecca Parales.[1] The plasmid pIP107D harboring the genes for CDO oxygenase α- (CumA1) and β- (CumA2) subunits, ferredoxin (CumA3) and reductase (CumA4) from *Pseudomonas fluorescens* IP01 was obtained from Hideaki Nojiri (Table S2).[2] Standard vectors were employed for cloning of all constructs (pET28a(+), pJET1.2, pUC118). CumA4 was inserted in pJET1.2 for amplification of the gene.

Table S2: Plasmids containing NDO and CDO system.

Construct	Vector	Resistance	Insert
**pUC19**	-	Amp	empty
**pDTG141**[1]	p11-based	Amp	NahAa, NahAb, NahAc, NahAd
**pIP107D**[2]	pUC-based	Amp	CumA1 WT, CumA2, CumA3 and partial CumA4
**pCDO**	pUC-based	Amp	CumA1 WT, CumA2, CumA3 and CumA4
**pCDOv1**	pUC-based	Amp	pCDO, with M232A mutation in CumA1

All primers were obtained from IDT (integrated DNA technologies, Inc.). A full list of primers can be found in Table S3. For amplification of DNA fragments, primers were designed with an average length of ~20 nucleotides (nt) and a melting temperature (Tm) difference of less than 5°C. The annealing temperature was calculated using the Tm Calculator from Thermo Fisher Scientific. For amplification of Gibson assembly® fragments, primers with a length of ~59 nt were designed. Gibson assembly primers contained a template binding region of ~20 nt and an overhang of ~40 nt. To fix a random mutation in pCDO and introduce a mutation at M232A in CumA1, primers were designed according to the Stratagene QuikChange™ protocol.

Table S3: List of primers used.

Name	Sequence 5'-3'	Description
**50_pIP107D_FW_seq_1**	GCCAATCTCCTCGGGACTTTGC	sequencing of pIP107D FW #1
**51_pIP107D1_RV_seq_1**	GCAAAGTCCCGAGGAGATTGGC	sequencing of pIP107D RV #1
**52_pIP107D_FW_seq_2**	GGATCCTTGTTGGTATGCATC	sequencing of pIP107D FW #2
**54_pIP107D_FW_seq_3**	CTTTAGGGTTCCGATTTAGTGC	sequencing of pIP107D FW #3
**55_pIP107D_cuma1_FW**	ATGAGTTCAATAATAAATAAAGAAG	sequencing of pIP107D FW CumA1
**56_pIP107D_cuma1_RV**	TCAAGACTTTAGCGTGTCCCAAC	sequencing of pIP107D RV CumA1
**57_pIP107D_cuma2_1_FW**	ATGACATCCGCTGATTTGACAAAAC	sequencing of pIP107D FW CumA2
**58_pIP107D_cuma2_2_RV_spacer**	GCAACTGTGCTTCGCGATAGTAG	sequencing of pIP107D RV CumA2 over spacer
**59_pIP107D_cuma2_3_FW_2**	GCTGGTACTTTGGAAGTTAGTTC	sequencing of pIP107D FW 2 (end of cuma2 and spacer)
**60_pIP107D_cuma3_1_FW**	ATGACTTTTTCCAAAGTTTGTG	sequencing of pIP107D FW CumA2
**61_pIP107D_cuma3_2_RV_spacer**	GTTGCGAACAACTCTCCATCGACG	sequencing of pIP107D RV CumA2 over spacer
**62_pIP107D_cuma3_3_FW_2**	GTGTCCGCACGGGCAAGGTAAAAG	sequencing of pIP107D FW 2 (end of cuma2 and spacer)
**63_pIP107D_cuma1_RV_spacer**	CTCATACAAATCCTGATCAGAG	sequencing of pIP107D RV CumA1 over spacer n-term
**64_pIP107D_cuma1_FW_spacer**	GCTAGAAGTAGACCTCTTTGTGC	sequencing of pIP107D RV CumA1 over spacer c-term
**65_pIP107D_FW_seq_4**	CTTCTGACAACGATCGGAGG	sequencing of pIP107D FW #4

**66_pIP107D_RV_seq_2_final**	CGTAGCTGGTCTTTCCAG	sequencing of pIP107D RV #2
**pDTG141 1_fwd**	GCCGACGAAATTGTCACTCAC	sequencing of pDTG141 for Reductase gene FW
**pDTG141 2_fwd**	GCGATGGTTGAAGCGTTG	sequencing of pDTG141 for Reductase gene FW
**pDTG141 3_fwd**	GGTAAGTGAATCTGGTCTGAGC	sequencing of pDTG141 for NDO α subunit FW
**pDTG141 4_fwd**	GCAAATGACCTCCAAATACGG	sequencing of pDTG141 for NDO α subunit FW
**pDTG141 5_fwd**	CTCCAACTGGGCTGAGTTC	sequencing of pDTG141 for NDO α and β subunit FW
**pDTG141 6_fwd**	AAGCATTGGTAACTGTCAGACC	sequencing of pDTG141 for NDO β subunit RV
**pDTG141 T7P_fwd**	TAATACGACTCACTATAGGG	sequencing of pDTG141 FW
**Primer_NDO_Cterm_fw**	CAATCTGATGGTCTTTCTGTG	sequencing of pDTG141 for NDO β subunit (C term) FW
**Amp_rv**	AGTGCTCATCATTGGAAAACG	sequencing of pDTG141 for Amp FW
**NDO_alpha_295A_forward**	CTCGGATTTATCGCAGCGCGCTCA ACTG	site directed mutagenesis on α subunit of NDO_FW
**NDO_alpha_295A_reverse**	GAAAACGGTGCAGTTGAGCGCGC TGC	site directed mutagenesis on α subunit of NDO_RV

RECONSTRUCTION OF PLASMID ENCODING CDO GENES

The plasmid pIP107D that was obtained from Hideaki Nojiri lacked a part of the reductase gene (CumA4). The reconstruction of CumA4 was performed by Gibson assembly®. The Gibson assembly fragments were amplified by a classical polymerase chain reaction (PCR). Reactions were set up as described in Tables S4-S7. The backbone was amplified using pIP107D as template and using Phusion High-Fidelity DNA Polymerase. The CumA4 insert was amplified using the native CumA4 gene as template and was amplified with DreamTaq DNA Polymerase. After synthesis, 3 PCR reactions were pooled to a final volume of 150 µL and digested using 3 µL *Dpn*I and incubated at 37°C for 2 h, followed by a heat inactivation at 80°C for 10 min. After *Dpn*I digest, the DNA concentrations of the fragments were measured using a NanoDrop™ 2000/2000c Spectrophotometer (Thermo Fisher Scientific). For the assembly, an equimolar amount of DNA was added to 15 µL assembly mix (final volume 20 µL) and incubated for 1 h at 50°C. At least 100 ng of the smallest fragment was used in the mix. After assembly, 3 µL of the assembly mix were used for transformation of 50 µl chemically competent *E. coli* TOP10 or Mach1 cells and plated on LB agar (1.5%) supplemented with 100 µg/mL ampicillin. The correct reconstruction of CumA4 was confirmed by DNA sequencing (primers given in Table S3) after isolation of the plasmids from overnight cultures of grown colonies. The final vector map of pCDOv1 is given in Figure S1.

Table S4: DreamTaq PCR mix.

DreamTaq	final volume 50 µL
5 µL	10x DreamTaq Buffer
1 µL	dNTP mix, 10 mM each
2.5 µL	FW primer (10 µM)
2.5 µL	RV primer (10 µM)
1 µL	Template
0.25 µL	DreamTaq DNA pol. (0.625 U)
to 50 µL	ddH_2O

Table S5: DreamTaq PCR Program.

PCR program	temperature	time
initial denaturation	95°C	1 min
30 cycles:		
denaturation	95°C	30 s
annealing	55 - 72°C	30 s
extension	72°C	1 min
final extension	72°C	1 min

Table S6: Phusion PCR mix.

Phusion	final volume 50 µL
10 µL	5x Phusion HF buffer
1 µL	dNTP mix, 10 mM each
2.5 µL	FW primer (10 µM)
2.5 µL	RV primer (10 µM)
1 µL	template
0.5 µL	Phusion DNA pol.
to 50 µL	ddH_2O

Table S7: Phusion PCR Program.

PCR program	temperature	time
initial denaturation	98°C	30 s
30 cycles:		
denaturation	98°C	10 s
annealing	66°C	30 s
extension	72°C	150 s
final extension	72°C	10 min

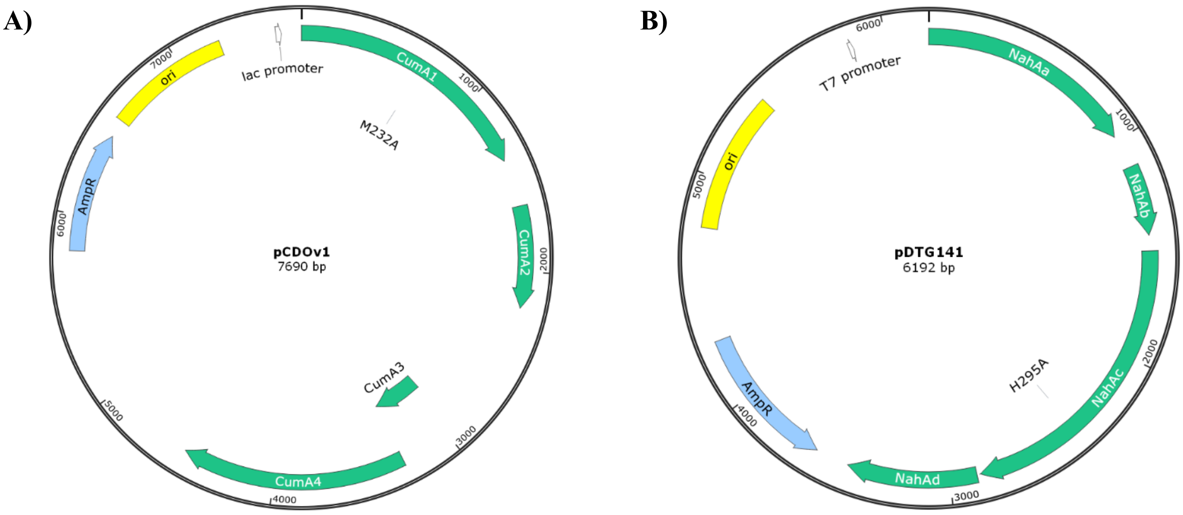

Figure S1: a) Plasmid pCDOv1 encoding the RO genes for CDO. pCDOv1 is based on the pIP107D vector (pUC18 backbone) containing an ampicillin resistance (AmpR) and genes are under the control of the IPTG-inducible *lac* promotor (lacP). **b)** Plasmid pDTG141 encoding the RO genes for NDO. pDTG141 is based on the p11 vector containing an ampicillin resistance (AmpR) and genes are under the control of the IPTG-inducible T7-promotor.

Site-directed mutagenesis

To fix a random mutation in CumA1 (G205V) and to introduce the mutation reported by Gally et al. (M232A),[3] site-directed mutagenesis was performed according to the Stratagene QuikChange™ protocol. Each QuikChange™ was performed with the respective primers listed in Table S3. A typical PCR mix (50 µl) consisted of 10x Pfu DNA polymerase buffer (5 µl), 1.25 µl of a mixture of deoxynucleoside triphosphates (2 mM each), Pfu DNA polymerase (0.625 U), 0.2 µL plasmid DNA (10 ng), and the forward and reverse primers (Table S3, 10 µM). After synthesis of the mutated strands, the methylated parental strands were digested using *Dpn*I. For the digestion with *Dpn*I, 1 µL was added to 50 µL PCR product and incubated at 37°C for 2 h, followed by a heat inactivation at 80°C for 10 min. The reaction mixtures were transformed in 50 µl chemically competent *E. coli* TOP10 or Mach1 cells and plated on LB agar (1.5%) supplemented with 100 µg/mL ampicillin. The correct introduction of the point mutations was confirmed by DNA sequencing (primers given in Table S3) after isolation of the plasmids from overnight cultures of grown colonies.

Expression of RO genes in *E. coli*

Heterologous expression of CDO and NDO was performed in *E. coli* JM109 (CDO) and *E. coli* JM109 (DE3) (NDO), respectively. 50 µL of chemo-competent *E. coli* cells were transformed with 2 µL plasmid DNA and incubated for 30 min on ice. After a heat shock at 42°C for 42 s, 400 µL LB-SOC was added and the cells were incubated at 37°C for 1 h. Finally, the cells were used to inoculate 5 mL liquid culture, and streaked out on LB plates, containing ampicillin (100 µg/mL). From the obtained colonies over-night cultures (ONCs) were prepared in 15 mL or 50 mL falcons by inoculating 5-10 mL LB medium, containing ampicillin at a final concentration of 100 µg/mL. Inoculation was performed with a colony obtained from an agar plate or 5 µL of a glycerol stock. After 16 h at 37°C, the ONC was used to inoculate 200 or 400 mL of TB/LB/ZYP-5052 medium to an initial OD_{600} of 0.1. The cultures were incubated at 37°C. After reaching an OD_{600} of 0.6 - 0.8, protein expression was induced with IPTG at a final concentration of 0.2 mM. After induction, the cells were incubated at 30°C. Cells were harvested by ultracentrifugation (1344 x *g*, 15 min, 4°C) 19 h after induction, washed with either sodium phosphate buffer (0.05 M, pH 7.2) or 50 mM MES and 50 mM MOPS buffer, respectively, and centrifuged again with the same speed. Cells were immediately used for *in vivo* biotransformations to avoid loss of activity. For generated variants, expression conditions of the wild type enzymes were used. For a qualitative analysis of cells expressing active ROs, the formation of indigo was observed by addition of indole. The assay was performed as described by Gally et al.[3] A Whatman filter paper was bathed in a 10% solution of indole in acetone. After the filter was dry it was placed in the lid of an agar plate containing *E. coli* colonies and incubated at room temperature, until indigo formation was observed.

We confirmed that all components were produced in a soluble form within the cytoplasm of *E. coli* (Figure S2). While the α-and β-subunits of CDO showed good expression in *E. coli*, the Fd and β-subunit of NDO were hardly visible on the SDS gel. Though, we have routinely examined the activity of the expressed RO systems by an agar plate assay based on indigo formation and could confirm significant activity toward indole (Figure S3 and S4).

SDS-PAGE

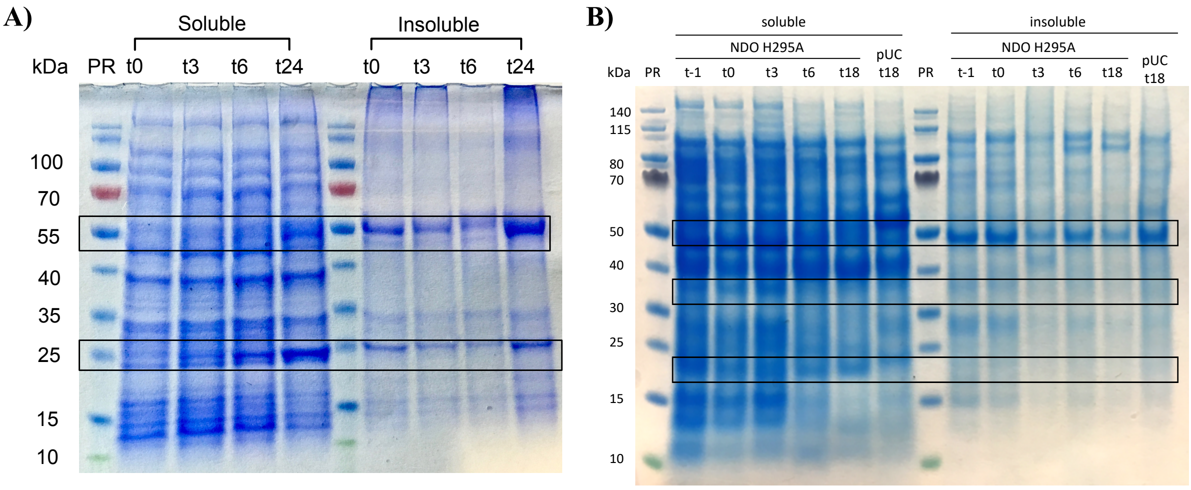

Figure S2: a) SDS-PAGE (Tris-Glycine) of *E. coli* JM109 harboring CDO M232A. Weak bands for CumA1 (55.8 kDa) and CumA2 (26.9 kDa) can be detected. No bands representing Fd (15.3 kDa) and FdR (47.9 kDa) can be found. **b)** SDS-PAGE (10% Bis-Tris gel in MES buffer) of *E. coli* JM109 (DE3) harboring NDO H295A. Weak bands for NahAa (35.51 kDa) and NahAc (49.61 kDa) can be detected. No bands representing NahAb (11.45 kDa) and NahAd (22.94 kDa) can be found. Protein standard: PageRuler Protein Ladder (Thermo Fisher Scientific).

AGAR PLATE ASSAY BASED ON INDIGO FORMATION

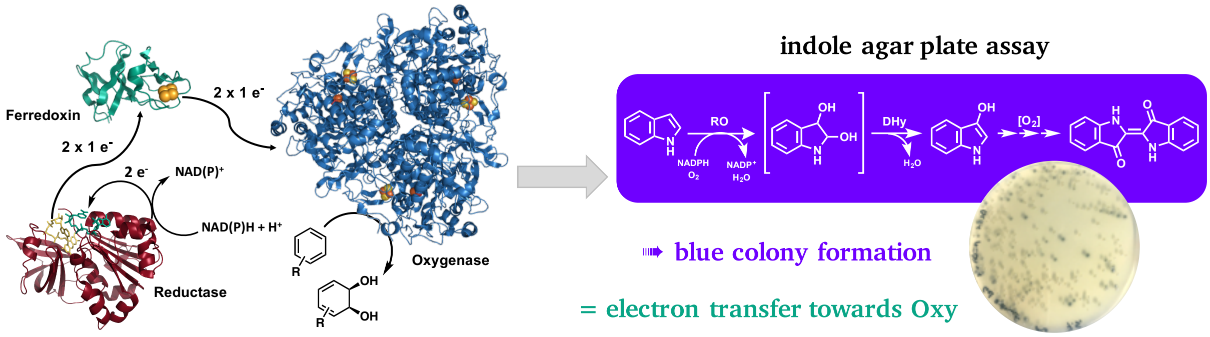

Figure S3: Solid phase colorimetric assay based on indigo formation. Due to the successful electron transport from the reductase via the ferredoxin and finally to the terminal oxygenase, the RO system catalyses the conversion of indole (from the tryptophan biosynthesis) to indigo within the *E. coli* cells. The assay confirmed successful overexpression and thus activity of the employed RO systems.

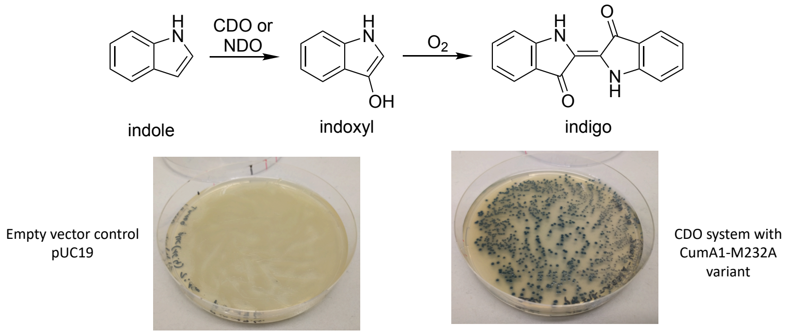

Figure S4: Solid phase colorimetric assay based on indigo formation used to determine activity of *E. coli* cells harboring CDO or NDO.

Biotransformations - Dark

In vivo and *in vitro* biotransformations of (*R*)-(+)-limonene, toluene and indene utilizing either cell-free extract or whole-cells were performed. First, *E. coli* cells harboring the respective construct encoding NDO and CDO, respectively, were cultivated and heterologous expression of the ROs was performed as described above. 19 h after induction, the cells were harvested and washed with sodium phosphate buffer (SPB, 50 mM, pH 7.2). First, the cultures were centrifuged for 20 min at 1344 x *g*. Washing was performed with 30 – 50 mL SPB, followed by an additional centrifugation step for 20 min at 1344 x *g*. Finally, the cells were resuspended to a wet cell weight (WCW) of 200 g_{WCW}/L in SPB for further use. The reactions performed with lysate were supplemented with NADPH as a cofactor, whereas, biotransformations performed with whole cells were supplemented with glucose. Table S8 gives an overview about the set-up of the dark reactions. 10 mM of the respective substrates were used. Reactions were incubated at 30°C and 180 rpm for up to 24 h. As negative control, *E. coli* JM109 harboring either the empty vector pUC118 as well as pIP107D harboring the incomplete CumA4 gene were used. An initial sample of 500 µL was taken at t_{0h}. For the determination of reaction kinetics, samples after 1, 2, 4, 6, 18, 24 h were taken from the reaction mixture and transferred in Eppendorf tubes, centrifuged and after addition of NaCl and acetophenone as internal standard, 500 µL supernatant was extracted 500 µL dichloromethane. The organic phase was dried over anhydrous magnesium sulfate.

Table S8: Cofactor supplemented reaction scheme for biotransformations in the dark.

Function	Component	Final concentration
**catalyst**	cell-free extract	250 µL/mL
	whole cells	10 - 200 g_{WCW}/L
	lyophilized cells	5 mg/mL
**cofactor**	NADPH	50 mM
	glucose	20 mM
**substrate**	(*R*)-(+)-limonene	
	indene	10 mM
	toluene	

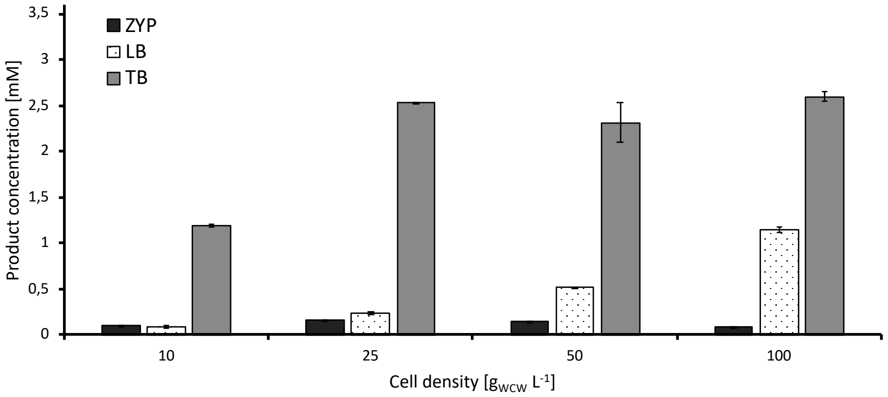

Figure S5: Biotransformations performed in the dark using the CDO system expressed in different media (autoinduction medium ZYP-5052, black; TB medium, grey; LB medium, with dotted) and by using different cell densities (final 10-100 g_{WCW}/L). Reaction conditions: 20 mM Glc, 10 mM (*R*)-limonene, 10-100 g_{WCW}/L whole cells (*E. coli* JM109_pCDOv1, 19h expression), sodium phosphate buffer pH 7.2, 30°C, 140 rpm, 24 hours.

BIOTRANSFORMATIONS - LIGHT

Whole cells harboring either NDO or CDO as well as cell free extracts have been prepared as described above. Light-driven reactions were examined using cell-free extract and whole-cells. Eosin Y (EY), 5(6)-carboxyeosin (CE), rose bengal (RB) and safranin O (SO) were investigated as photosensitizer. Furthermore, ethylenediaminetetraacetic acid (EDTA), 3-morpholinopropane-1-sulfonic acid (MOPS) and 2-(N-morpholino)ethanesulfonic acid (MES), respectively, were supplemented as electron donor. Excitation was performed using white or blue light. Light-driven reactions, supplemented with photosensitizers and electron donors, were performed at 30°C in the 3D printed light reactor with LED light (Figure S6) or a homemade light-reactor placed in an incubation shaker (under gentle agitation) equipped with fluorescent tubes (Figure S7). Reactions without light excitation, which were supplemented with cofactors (NADPH / glucose), were performed in a dark incubation shaker at 30°C. Biotransformations were set up as seen in Table S9 at a total volume of 1 in 1.5 mL screw capped glass vials or 2 in 20 mL screw capped glass vials due to the volatility of the tested substrates. 10 mM of the respective substrates were used. Reactions were incubated at 30°C and 180 rpm for up to 72 h. As negative control, *E. coli* JM109 or *E. coli* JM109 (DE3) cells harboring either the empty vector pUC118 were used. An initial sample of 500 µL was taken at t_{0h}. For the determination of reaction kinetics, samples after 1, 2, 4, 6, 18 and 24 were taken from the reaction mixture and transferred in Eppendorf tubes, centrifuged and after addition of NaCl and acetophenone as internal standard, 500 µL supernatant was extracted 500 µL dichloromethane. The organic phase was dried over anhydrous magnesium sulfate.

Table S9: Light-driven reaction scheme.

Function	Component	Final concentration
**catalyst**	cell-free extract	250 µL/mL
	whole cells	25 - 300 g_{WCW}/L
	lyophilized cells	5 mg/mL
**photosensitizer**	EY	
	RB	100 µM
	CE	
	SO	
**electron donor**	EDTA	25 mM
	MOPS	50 mM
	MES	50 mM
**substrate**	indene	
	(*R*)-(+)-limonene	10 mM
	toluene	

<u>PHOTOCATALYTIC SETUP</u>

3D printed light reactor

A 3D printed light reactor was created for the implementation of light-driven biotransformations in 20 mL glass vials. Using Fusion 360 (Autodesk Inc.), a model was designed. The designed model was 3D printed using the Ultimaker2 (Ultimaker B.V.) with polylactide (PLA). The interior of the light reactor was lined with conventional LED strips (RGB, 12 V). The 3D printed light reactor was placed in an incubator shaker at 30°C and 130 rpm.

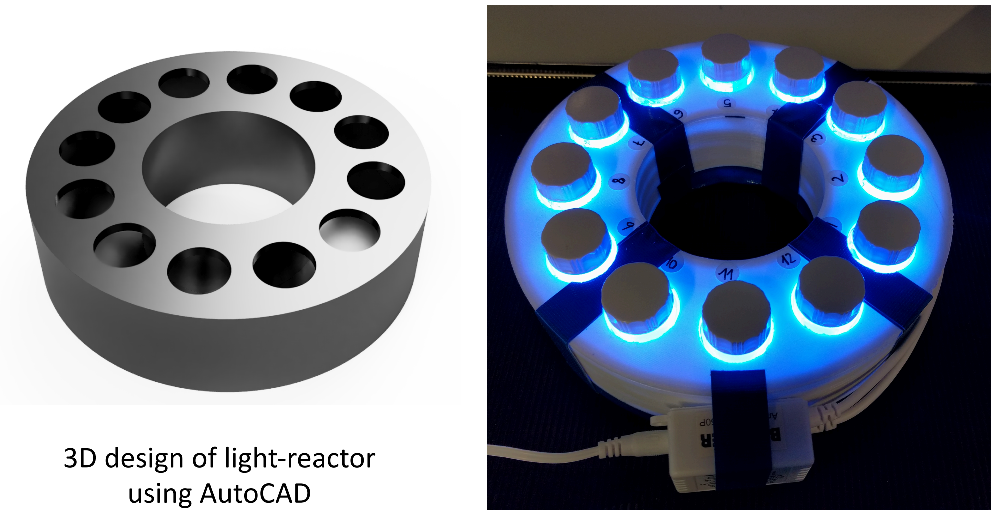

Figure S6: 3D printed light reactor equipped with LED stripes. Left: 3D concept of light reactor, made with autocad. Right: Final 3d printed light reactor holding 20 mL vials and lined with led strips

Fluorescent lamp light reactor

The homemade fluorescent lamp containing light reactor (Figure S7) was also dedicated for biotransformations in 20 mL glass vials but consisted of three fluorescent lamps instead of LED stripes. This light reactor was also placed in an incubation shaker at 30°C with 130 rpm agitation.

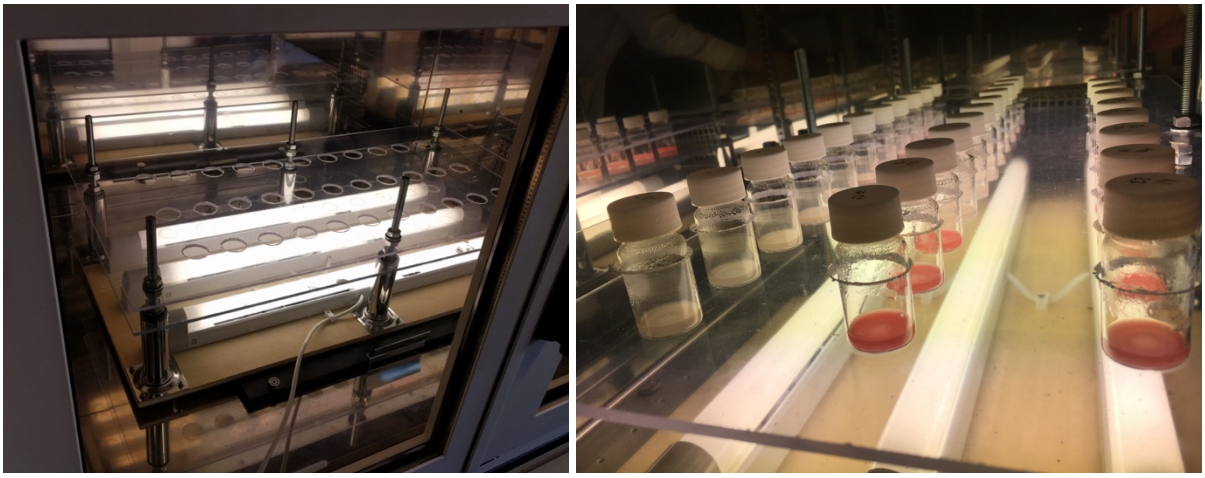

Figure S7: Light reactor equipped with fluorescent lamps which is placed into an incubator.

Absorption spectra of used fluorescent dyes as photosensitizer

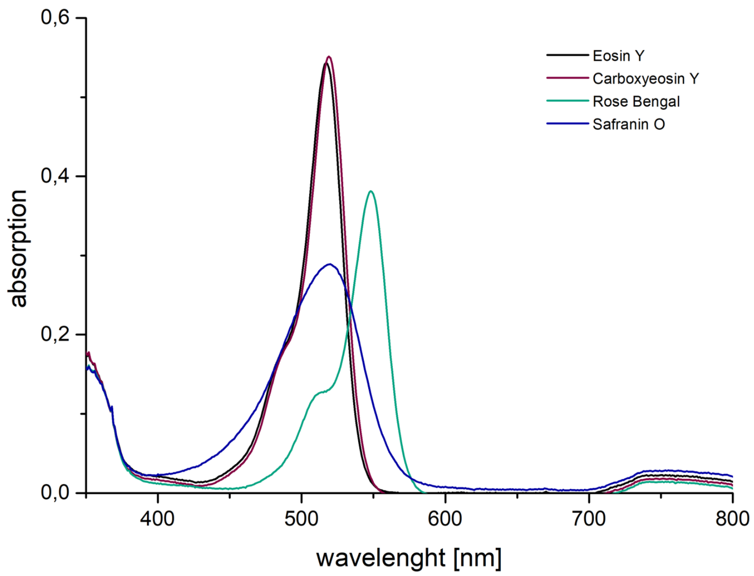

Figure S8: Absorption spectrum of eosin Y, carboxyeosin Y, rose bengal and safranin O determined from 350 to 800 nm in sodium phosphate buffer (pH 7.2, 100 mM).

EFFECT OF LIGHT INTENSITY ON PRODUCT FORMATION DURING WHOLE-CELL BIOTRANSFORMATIONS WITH NDO_H295A

Due to the experimental setup used for the light-driven whole-cell biotransformations, variations in reaction conditions and thus product formation rate could be observed. Especially the variation of light intensities attributable to the light-reactor design was supposed to have a significant impact on product formation. The fluorescence light-reactors used in this work were designed to establish almost constant reaction conditions in terms of either temperature and bottom-up light exposure. However, light exposures at relevant positions within the reactors (see Figure S7, emission spectrum see Figure S11A) show noticeable differences which were determined by a light measure device (Figure S9 and S10) and by chemical actinometry (Figure S11B, experimental details see below). To evaluate a possible correlation between light exposure and product formation in the course of the light-driven whole-cell biotransformations, the conversion of 10 mM indene catalyzed by *E. coli* JM109 (DE3) cells harboring NDO_H295A at different light intensities was followed (Figure S9A and B). Therefore, 1 mL reaction mixtures were prepared with final concentrations of 100 g_{WCW} L^{-1} resting *E. coli* cells resuspended in 50 mM MES buffer. As a photosensitizer, CE at a final concentration of 100 µM was added. After 24 h incubation at 28 °C, the product concentrations gained at certain light intensities were determined by GC-MS. To extend the data range, analogous experiments with dimmed light sources (dimmed by around 75% per position) were also conducted. The detected product concentrations are plotted against the respective light intensities (Figure S9B). For the sake of better interpretation, a Hill fitting model was calculated by OriginLab software based which is indicated as red curve.

A) B)

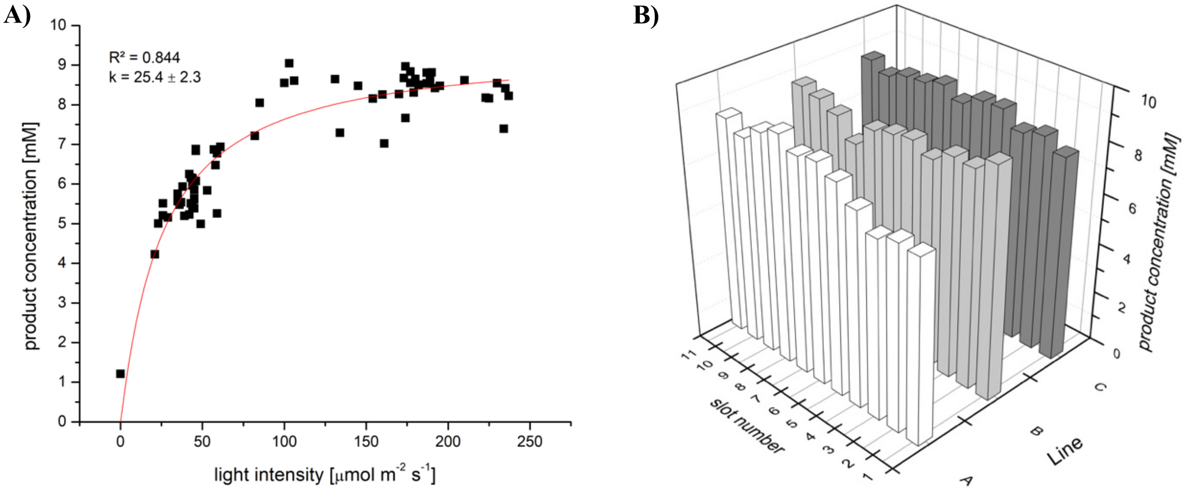

Figure S9: A) Scatter plot displays of product concentrations obtained at different light intensities in whole cell biotransformations using 10 mM of **2** catalyzed by NDO_H295A. The red curve represents a Hill model fitted to the data by OriginLab software. Abscissa: light intensity in µmol m^{-2} s^{-1}; ordinate: product concentration in mM. **B)** Product concentrations per position in the light-reactor.

A) B)

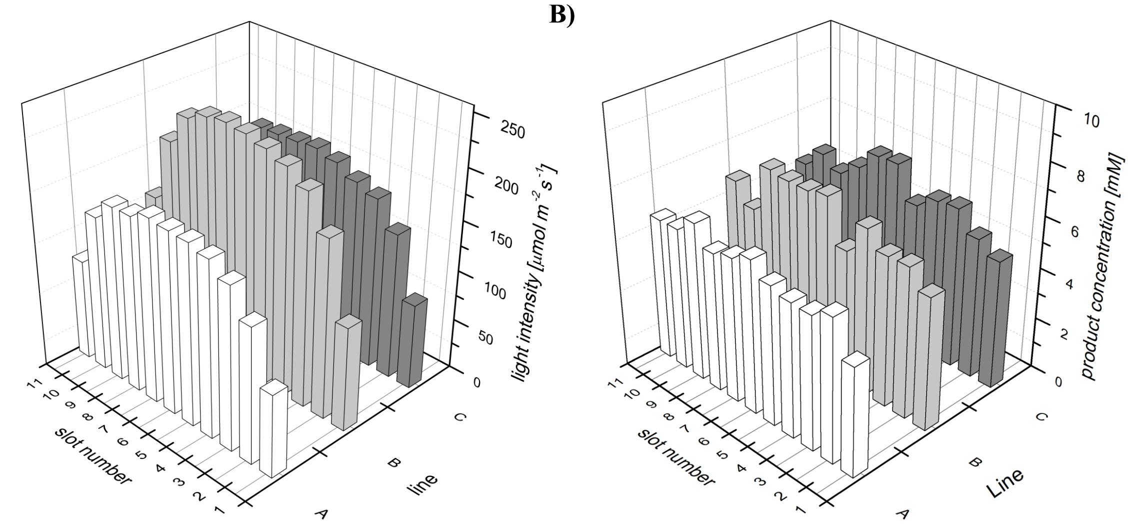

Figure S10: A) Determined light intensities of fluorescence light reactor. Line and slot number refer to the actual local position within the light reactor. Values for light intensities were determined with a light measure device and depicted as amount of photons count per square metre per second [µmol m^{-2} s^{-1}]. **B)** Obtained product concentrations when light intensities were decreased by 75% in each position of the light reactor.

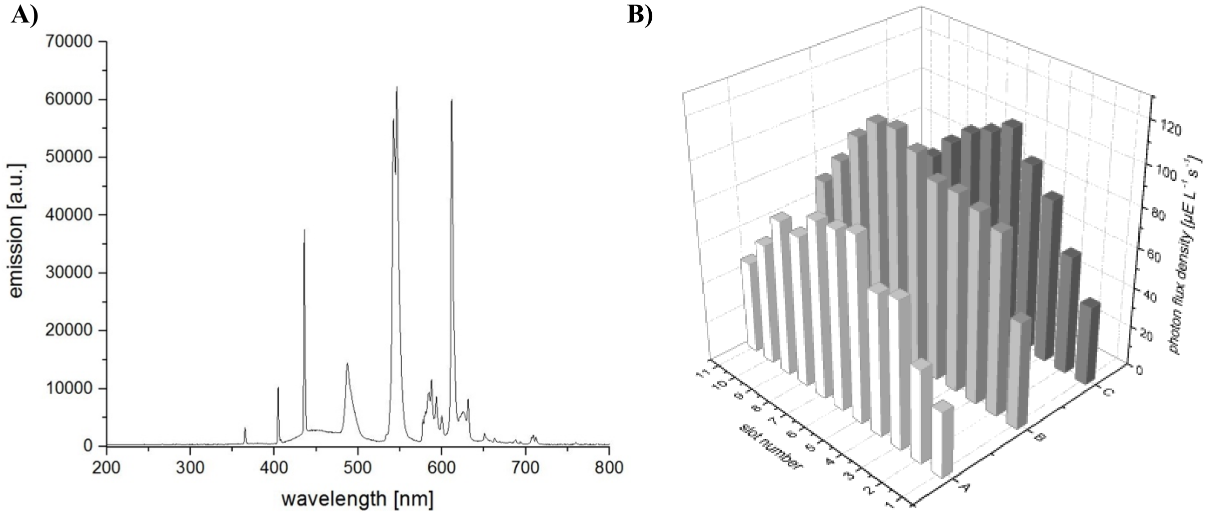

Figure S11: A) Determined emission spectrum of the fluorescent lamps used in the fluorescence light reactor. **B)** Photon flux densities [µE $L^{-1}s^{-1}$] in each position of the fluorescence light reactor determined by chemical actinometry.

Actinometry and quantum yield determination

Chemical actinometry to determine the incident photon flux was performed using the well-described ferrioxalate actinometer.[6] Briefly, 1 mL of a 0.15 M solution of freshly prepared potassium ferrioxalate was put into the same vials used for the other experiments and irradiated in the same setup. The mean optical pathlength in this setup was calculated to be 0.456 cm. Over the course of 50 s, the concentration of Fe(II) formed was determined every 10 s photometrically using the ferroin-complex and a linear formation rate was calculated (R_{Fe}). The incident photon flux (q_p) was then calculated according to eqn. X1 from this Fe(II) formation rate by considering the spectrum of the lamp on a number of photon basis ($E_p(\lambda)$), the transmission of the ferrioxalate solution ($T(\lambda)$) (both measured using an Ocean Optics FLAME-S-UV-VIS-ES spectrophotometer) as well as the known quantum yield of Fe(II) formation ($\Phi(\lambda)$)[6] and integrating over the whole wavelength region of interest (200 to 800 nm).

$$q_p = R_{Fe} \cdot \frac{\int E_p(\lambda)\, d\lambda}{\int E_p(\lambda) \cdot \Phi(\lambda) \cdot \left(1 - T(\lambda)\right) d\lambda} \qquad \text{(X.1)}$$

Apparent quantum yields (AQY) were then determined according to eqn. X.2 by calculating the ratio of two times the observed product formation rate (R) to the incident photon flux, as two photons are required per turnover.

$$AQE = \frac{2 \cdot R}{q_p} \qquad \text{(X.2)}$$

Moreover, it was attempted to estimate the true quantum yield (QY) of the reaction. Due to strong scattering in the cell suspension as well as optical absorption by other cell or solution components, this is a very challenging task. The numbers presented here should therefore only

be considered as a very rough estimate. By measuring the absorption spectra of the cell suspension with and without the respective dye (Figure S12), the (optically) effective dye concentration was estimated from the difference of the two considering the extinction coefficients of the dyes. In the next step, the fraction of the light from the lamp that is absorbed by the dye was calculated by using the lamp spectrum as well as the dye absorption spectrum at its effective concentration and again integrating over the whole wavelength region of interest (200 to 800 nm). Estimated quantum yields were then calculated by dividing the apparent quantum yield values by the fraction of light absorbed by the dye.

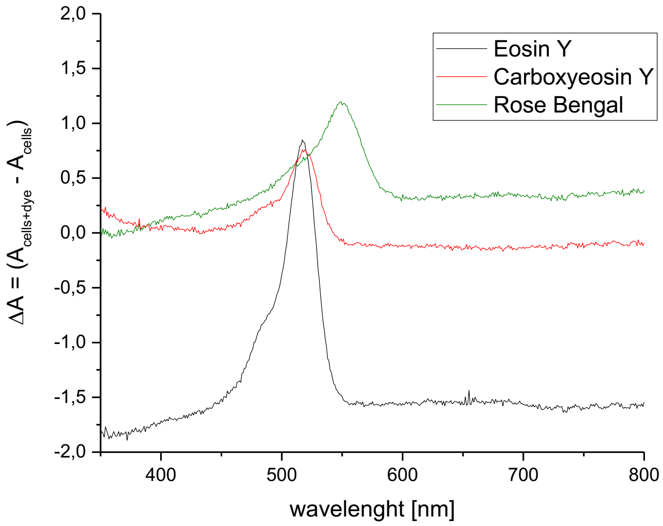

Figure S12: Differential absorption spectra of the cells containing the dyes eosin Y, 5(6)-carboxyeosin and rose bengal and the cells without the dyes.

Apparent quantum yield has been calculated with the maximum photon flux density and can therefore be considered as a lower limit for the AQY.

Table S10: Apparent quantum yields and estimated quantum yields for the light-driven hydroxylation catalyzed by CDO M232A or NDO H295A with different photosensitizer/electron donor combinations (corresponding to Table2).

**Enzyme**	**Electron Donor**	**Photosensitizer**	**Rate [mM/h]**	**AQY [%]**	**QY [%]**
CDO M232A	EDTA	EY	0.18	0.09	0.37
	MOPS	EY	0.13	0.06	0.27
	MES	EY	0.25	0.12	0.51
	EDTA	RB	1.59	0.78	2.21
	MOPS	RB	0.94	0.46	1.31
	MES	RB	1.65	0.81	2.29
	EDTA	CE	0.26	0.13	0.99
	MOPS	CE	0.61	0.30	2.33
	MES	CE	0.75	0.37	2.86
NDO H295A	EDTA	EY	0.23	0.11	0.47
	MOPS	EY	0.37	0.18	0.76
	MES	EY	0.52	0.26	1.07
	EDTA	RB	0.32	0.16	0.44

	MOPS	RB	0.89	0.44	1.24
	MES	RB	0.45	0.22	0.62
	EDTA	CE	0.30	0.15	1.14
	MOPS	CE	0.86	0.42	3.28
	MES	CE	0.64	0.32	2.44

CELL VIABILITY STUDIES

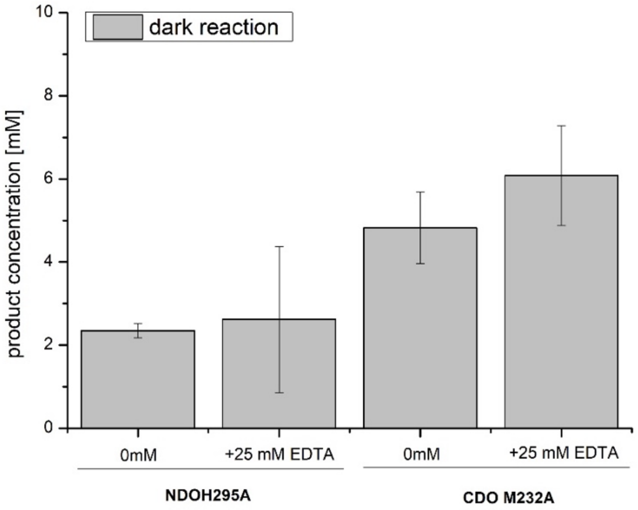

Figure S13: Performed control reactions using NDO H295A and CDO M232A under dark conditions to investigate a potential toxicity effect of EDTA on the cells. Reaction conditions: [substrate] = 10 mM indene, [glucose] = 20 mM, [whole cells] = 100 g_{WCW}/L (*E. coli* JM109 (DE3)_pDTG141_NDO_H295A or *E. coli* JM109_pCDOv1, 19h expression), sodium phosphate buffer (pH 7.2, 50 mM), 24 hours.

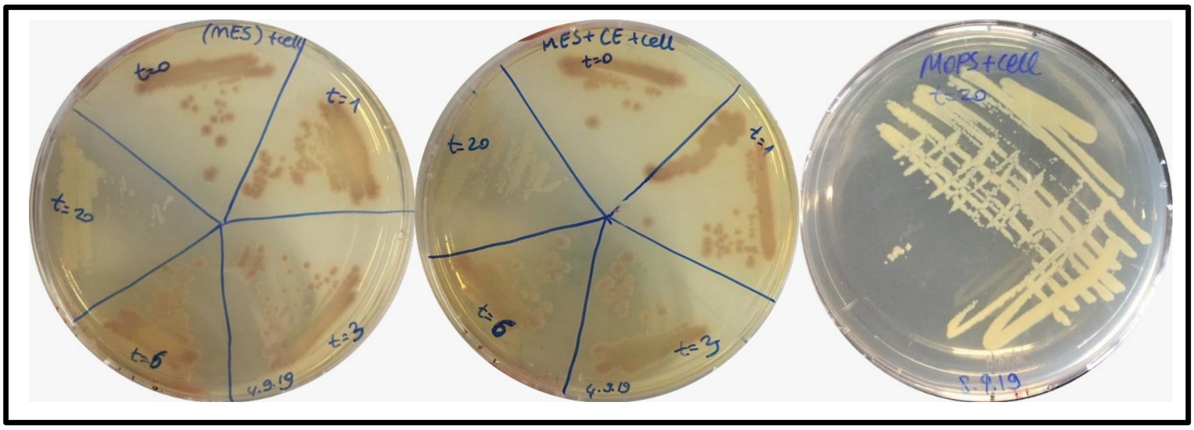

Figure S14: Streaking of *E. coli* JM109 (DE3) cells containing the construct pDTG141_NDO H295A after different time points during the light-driven biotransformations containing either MES (left), MES and CE (middle) and MOPS (right).

Table S11: Photobiocatalytic hydroxylation reactions catalyzed by cell lysate containing NDO variant H295A using CE as photosensitizer and MES as electron donor with different substrates.

Substrate	Reaction Conditions	Substrate Conc.	Product Conc. [mM]
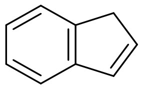	dark/20 mM NADPH<br>dark/CE/MES<br>light/ CE/ MES	10 mM	1.72 ± 0.03<br>0.16 ± 0.02<br>5.55 ± 0.1
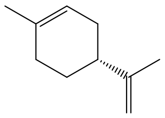	dark/20 mM NADPH<br>dark/CE/MES<br>light/ CE/ MES	10 mM	**Product Conc. [µM]**<br>30.1 ± 1.47<br>17.8 ± 0.70<br>57.7 ± 3.67

Reaction conditions dark: [NADPH] = 20 mM, [cell lysate] = 0.2 U/mL, sodium phosphate buffer (pH 7.2, 50 mM), 24 hours. Reaction conditions light: [cell lysate] = 0.2 U/mL, MES buffer (pH 7.2, 50 mM), CE = [100 µM] white light illumination (max. 112 µE L^{-1} s^{-1}) 24 hours.

<u>PRODUCT DISTRIBUTION INDENE</u>

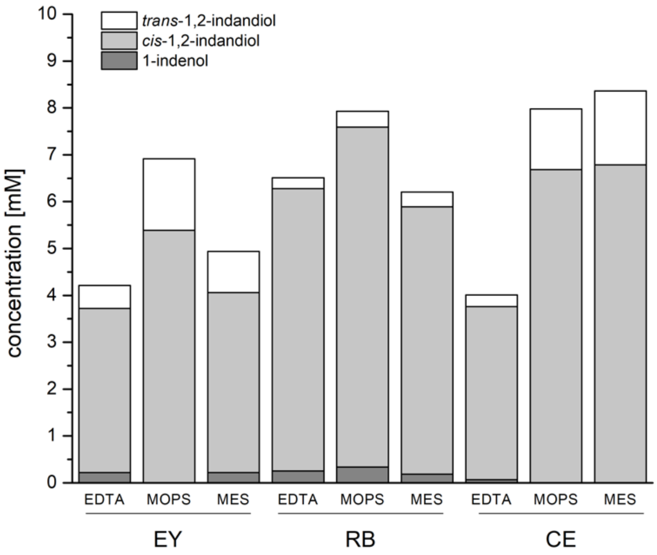

Figure S15: Product distribution in light-driven whole-cell oxyfunctionalization reactions catalyzed by CDO M232A determined after 24 hours. Reaction conditions: 100 µM photosensitizer, 10 mM indene **2**, 25 mM EDTA or 50 mM MOPS/MES, 100 g_{WCW}/L whole cells (*E. coli* JM109_pCDOv1, 19h expression), 50 mM SPB pH 7.2, white light (max. 112 µE L^{-1} s^{-1}), 30°C, 140 rpm, 24 hours.

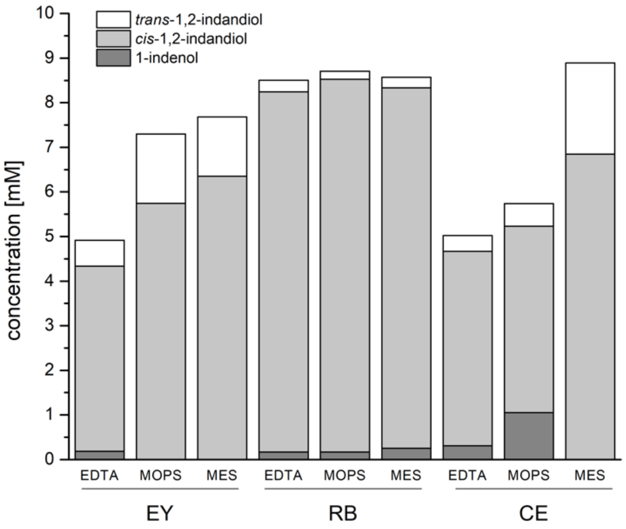

Figure S16: Product distribution in light-driven whole-cell oxyfunctionalization reactions catalyzed by NDO H295A determined after 24 hours. Reaction conditions: 100 µM photosensitizer, 10 mM indene **2**, either 25 mM EDTA or 50 mM MES/MOPS, 100 g_{WCW}/L whole cells (*E. coli* JM109 (DE3)_pDTG141_NDO_H295A, 19h expression), 50 mM SPB pH 7.2, white light (max. 112 µE L^{-1} s^{-1}), 30°C, 140 rpm, 24 hours.

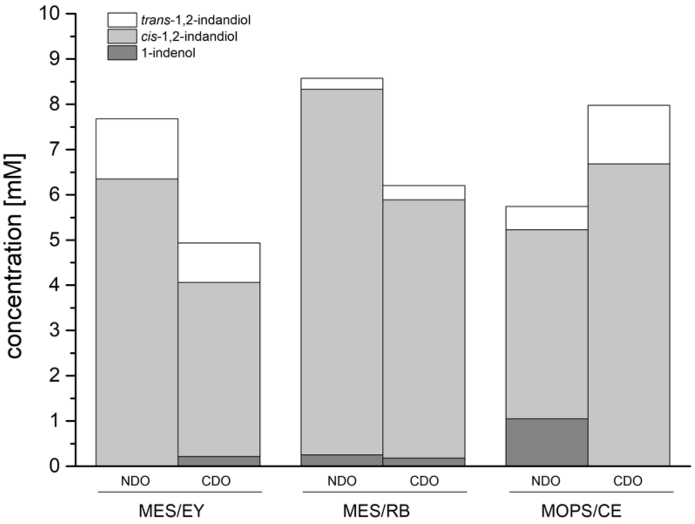

Figure S17: Product distribution in light-driven whole-cell oxyfunctionalization reactions catalyzed by NDO H295A and CDO M232A depicted as direct comparison for photosensitizer/electron donor combinations leading to the highest product formation. Reaction conditions: 100 µM photosensitizer, 10 mM indene **2**, either 25 mM EDTA or 50 mM MES/MOPS, 100 g_{WCW}/L whole cells cells (*E. coli* JM109 (DE3)_pDTG141_NDO_H295A or *E. coli* JM109_pCDOv1, 19h expression), 100 mM SPB pH 7.2, white light (max. 112 µE L^{-1} s^{-1}), 30°C, 140 rpm, 24 hours.

Table S12: Side-product formation during the photobiocatalytic hydroxylation catalyzed by ROs using different photosensitizer and electron donors and indene as substrate.

Enzyme	Photosensitizer Electron Donor	Substrate	Σ Side product(s)[a] [mM]	Concentration Quinoline [mM]	Concentration 1-indanone [mM]
CDO M232A	EY / EDTA		5.8	4.3	0.3
	EY / MOPS		3.0	2.1	0.9
	EY / MES		4.5	4.0	0.5
	RB / EDTA		3.3	2.7	0.6
	RB / MOPS		2.0	1.5	0.6
	RB / MES		3.1	2.9	0.3
	CE / EDTA		0.8	2.5	0.1
	CE / MOPS		1.9	1.8	0.1
	CE / MES	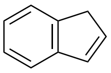	1.6	1.4	0.2
NDO H295A	EY / EDTA		4.7	4.4	0.3
	EY / MOPS		2.7	2.3	0.4
	EY / MES		2.0	1.5	0.6
	RB / EDTA		0.7	0.4	0.3
	RB / MOPS		0.6	0.4	0.2
	RB / MES		1.4	1.1	0.3
	CE / EDTA		2.3	2.1	0.2
	CE / MOPS		0.7	0.2	0.5
	CE / MES		1.1	0.9	0.2

[a] Mixture of quinoline (up to 35%) and 1-indanone (<5%) which are the main side-products observed, others were only formed in traces and were not quantified by GC.

Influence of Cell density and electron donor concentration on Light-driven Hydroxylation

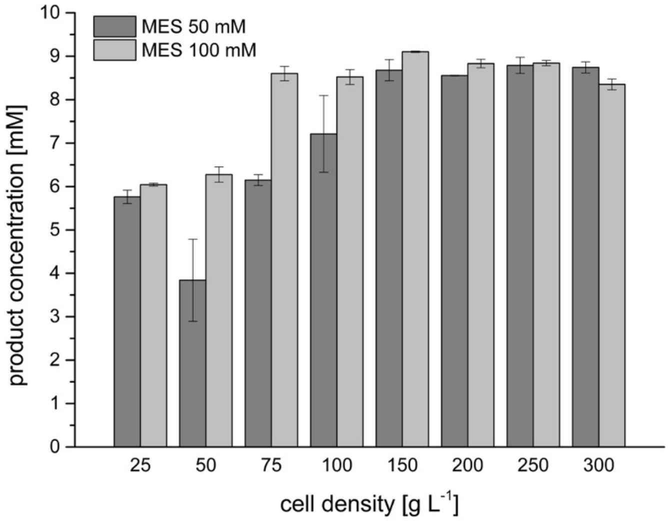

Figure S18: Effect of biocatalyst concentration and electron donor concentration in light-driven whole-cell hydroxylation reactions using CE catalyzed by NDO H295A and MES as electron donor. Reaction conditions: 100 µM photosensitizer, 10 mM indene **3**, 50/100 mM MES, in 25- 300 g_{WCW}/L whole cells (*E. coli* JM109 (DE3)_pDTG141_NDO_H295A , 19h expression), 50 mM SPB pH 7.2, white light (max. 112 µE L^{-1} s^{-1}), 30°C, 140 rpm, 24 hours.

Control Reactions

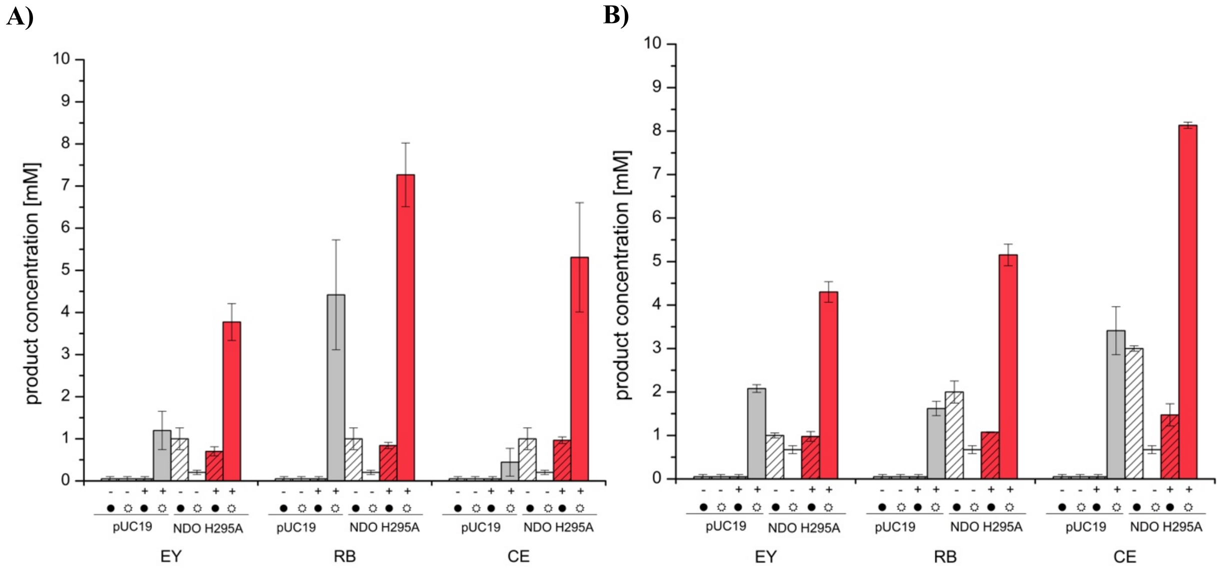

Figure S19: A) Performed control reactions using NDO H295A under light (☼) and dark (•) conditions with (+) or without (-) 100 µM EY, RB or CE in the presence of NDO H295A (red bars) or with an empty vector control (grey bars) with EDTA. **B)** Performed control reactions using NDO H295A under light (☼) and dark (•) conditions with (+) or without (-) 100 µM EY, RB or CE in the presence of NDO H295A (red bars) or with an empty vector control (grey bars) with MES. Reaction conditions: 100 µM photosensitizer, 10 mM **3**, 25 mM EDTA or 50 mM MES, 100 g_{WCW}/L whole cells (*E. coli* JM109 (DE3)_pDTG141_NDO_H295A, 19h expression), 50 mM SPB pH 7.2, white light (max. 112 µE L^{-1} s^{-1}), 30°C, 140 rpm, 24 hours.

Kinetic profiles of photobiocatalytic hydroxylations

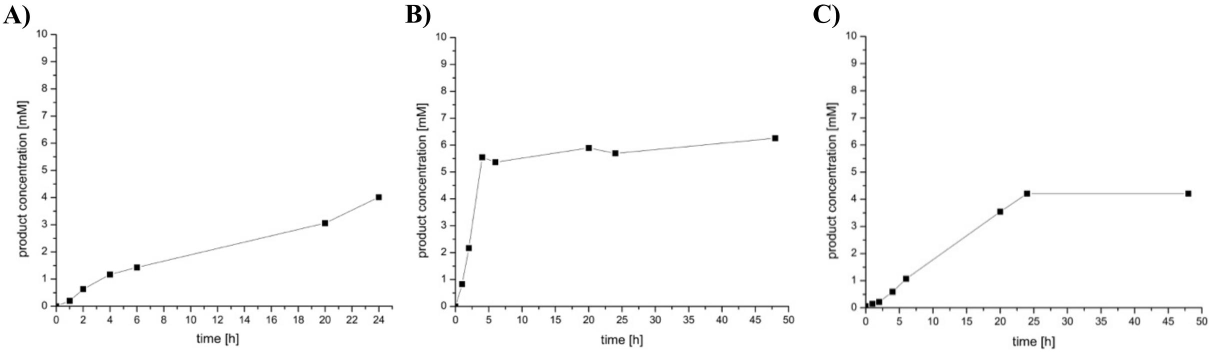

Figure S20: Kinetic profile obtained for the light-driven whole-cell hydroxylation reaction employing CDO M232A CE (A), RB (B) and EY (C) in combination with EDTA as electron donor. Reaction conditions: 100 µM photosensitizer, 10 mM **3**, 25 mM EDTA, in 100 g_{WCW}/L whole cells (*E. coli* JM109_pCDOv1, 19h expression), 50 mM SPB pH 7.2, white light (max. 112 µE L^{-1} s^{-1}), 30°C, 140 rpm, 24-48 hours.

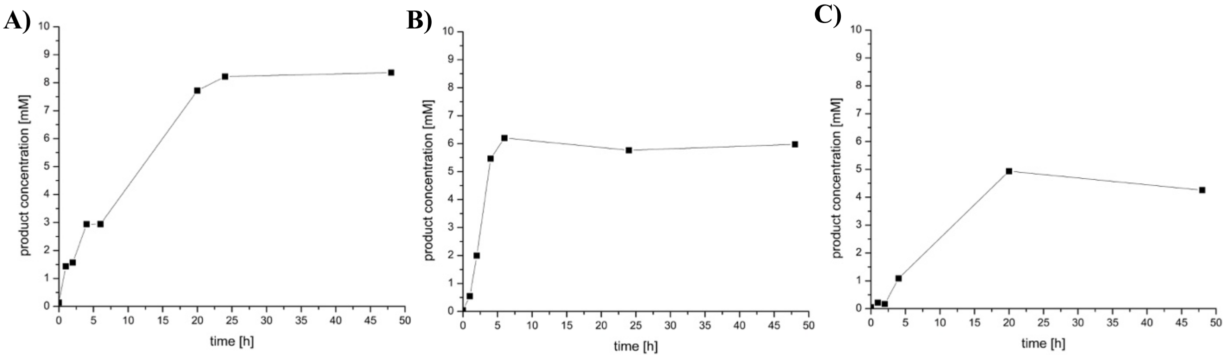

Figure S21: Kinetic profile obtained for the light-driven whole-cell hydroxylation reaction employing CDO M232A CE (A), RB (B) and EY (C) in combination with MES as electron donor. Reaction conditions: 100 µM photosensitizer, 10 mM **3**, 50 mM MES, in 100 g_{WCW}/L whole cells (*E. coli* JM109_pCDOv1, 19h expression), 50 mM SPB pH 7.2, white light (max. 112 µE L^{-1} s^{-1}), 30°C, 140 rpm, 48 hours.

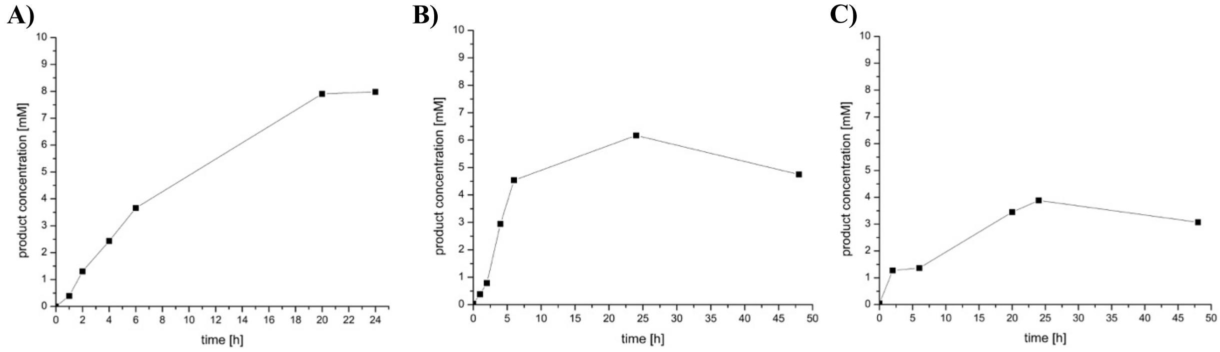

Figure S22: Kinetic profile obtained for the light-driven whole-cell hydroxylation reaction employing CDO M232A CE (A), RB (B) and EY (C) in combination with MOPS as electron donor. Reaction conditions: 100 µM photosensitizer, 10 mM **3**, 50 mM MOPS, in 100 g_{WCW}/L whole cells (*E. coli* JM109_pCDOv1, 19h expression), 50 mM SPB pH 7.2, white light (max. 112 µE L^{-1} s^{-1}), 30°C, 140 rpm, 48 hours.

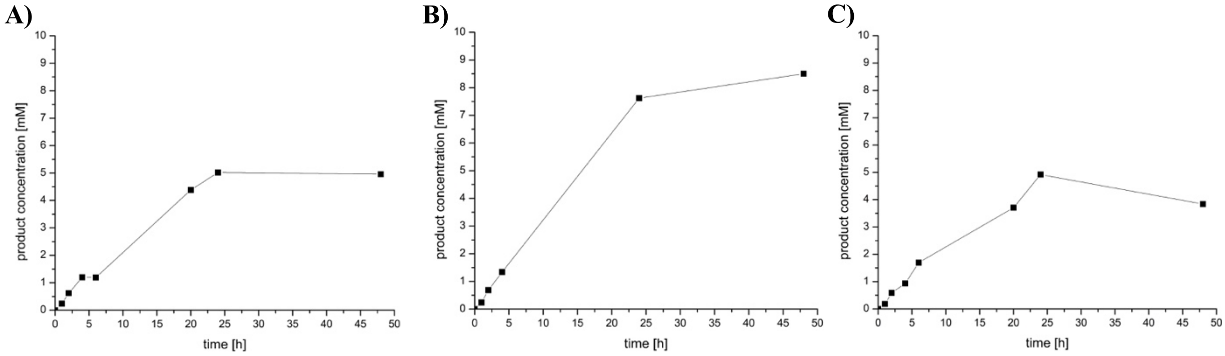

Figure S23: Kinetic profile obtained for the light-driven whole-cell hydroxylation reaction employing NDO H295A CE (A), RB (B) and EY (C) in combination with EDTA as electron donor. Reaction conditions: 100 µM photosensitizer, 10 mM **3**, 25 mM EDTA, in 100 g_{WCW}/L whole cells (*E. coli* JM109 (DE3)_pDTG141_NDO_H295A, 19h expression), 50 mM SPB pH 7.2, white light (max. 112 µE L^{-1} s^{-1}), 30°C, 140 rpm, 48 hours.

A) B) C)

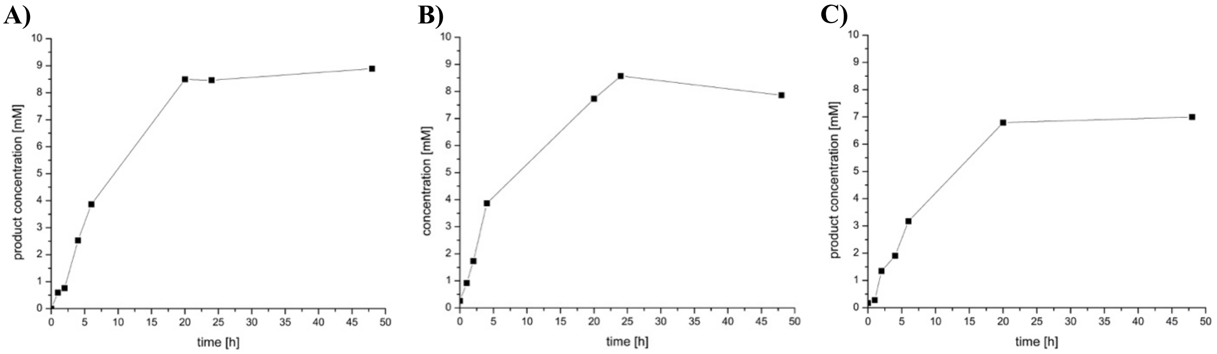

Figure S24: Kinetic profile obtained for the light-driven whole-cell hydroxylation reaction employing NDO H295A CE (A), RB (B) and EY (C) in combination with MES as electron donor. Reaction conditions: 100 µM photosensitizer, 10 mM **3**, 50 mM MES, in 100 g_{WCW}/L whole cells (*E. coli* JM109 (DE3)_pDTG141_NDO_H295A, 19h expression), 50 mM SPB pH 7.2, white light (max. 112 µE L^{-1} s^{-1}), 30°C, 140 rpm, 48 hours.

A) B) C)

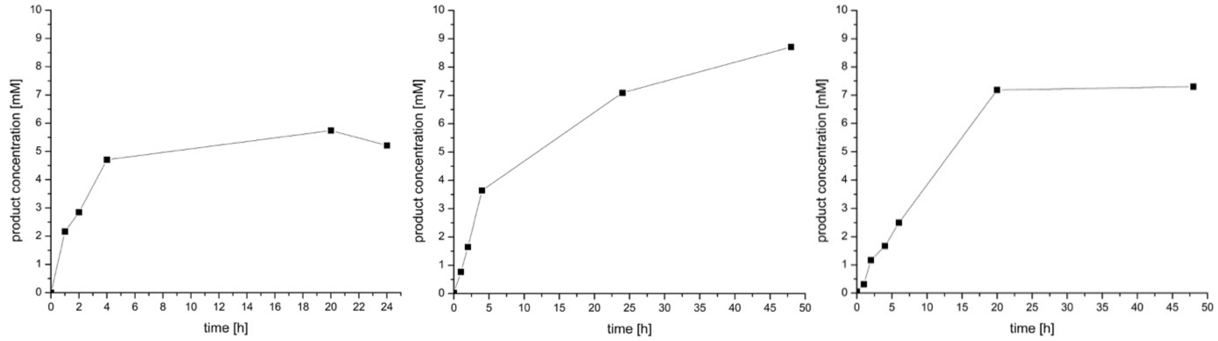

Figure S25: Kinetic profile obtained for the light-driven whole-cell hydroxylation reaction employing NDO H295A CE (A), RB (B) and EY (C) in combination with MOPS as electron donor. Reaction conditions: 100 µM photosensitizer, 10 mM **3**, 50 mM MOPS, in 100 g_{WCW}/L whole cells (*E. coli* JM109 (DE3)_pDTG141_NDO_H295A, 19h expression), 50 mM SPB pH 7.2, white light (max. 112 µE L^{-1} s^{-1}), 30°C, 140 rpm, 48 hours.

PHOTOBIOCATALYTIC HYDROXYLATION WITH LYSED CELLS

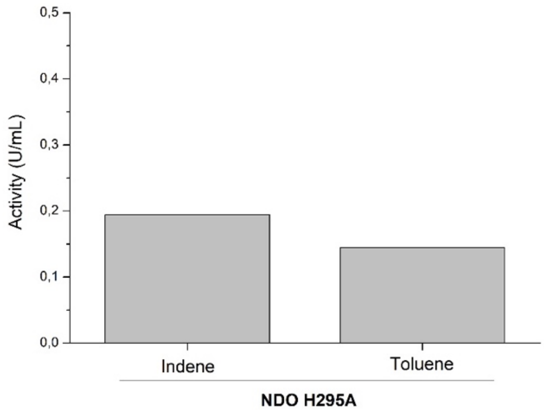

Figure S26: Spectrophotometric determination of volumetric activity of lysed cells containing NDO H295A against different substrates.

ANALYTICS

GC analysis

All measurements have been performed at least as duplicates. Numbers shown are always based on calibration curves with authentic standards of the reagents (using internal standards). Error bars shown represent the standard deviation.

In general, samples were taken from the reaction mixtures at defined time intervals as described above. Organic phase extraction of the biotransformation samples was performed using dichloromethane (DCM). First, a spatula tip of NaCl was added to 500 µL sample and briefly vortexed. The sample was extracted with 500 µL DCM containing 2 mM acetophenone. Extraction was performed by adding DCM, vortexing the mixture for 10 s, followed by a centrifugation step for 2 min at 16000 *g*. The lower phase was transferred to an Eppendorf tube containing a spatula tip of $MgSO_4$. After vortexing for 10 s, the samples were centrifuged for 10 min at 16.000 *g*. After centrifugation 200 µL sample was transferred to a 2 mL GC-vial, containing a 250 µL inlet. Finally, 1 µl sample was injected into the GC instrument.

GC-FID

Achiral quantitative analysis was performed on a Shimadzu GC-2010 Plus with a Zebron ZB-5 capillary column with nitrogen as carrier gas. Chiral quantitative analysis was performed on a Shimadzu Nexis GC-2030 equipped with a Hydrodex-β-6TBDM capillary column. For each substrate and respective product, a method was established. This enabled the detection of (*R*)-(+)-limonene and carveol in the established method. Parameters and GC temperature programs can be seen in Tables S13-S16.

Table S13: GC-FID parameters used for achiral analytics of (*R*)-(+)-limonene and products.

**Achiral GC-FID parameters – (*R*)-(+)-limonene detection**	
Instrument	GC-2010 Plus (Shimadzu Europa GmbH.)
Column	Zebron ZB-5 (Phenomenex Inc.)<br>length: 30 m, inner diameter: 0.32 mm, film thickness: 0.25 µM
Injection volume	1 µL
Injection temp.	230°C
Injection mode	Split
Flow control mode	pressure
Pressure	22.7 kPa
Total flow	15.6 mL / min
Column flow	0.6 mL / min
Linear velocity	12.5 cm / s
Purge flow	3 mL / min
Split ratio	20
Oven temp. program	4 min at 60°C, 5°C min^{-1} to 165°C, 2 min at 165°C, 15°C min^{-1} to 300°C, 2 min at 300°C
FID temperature	320°C
Compound retention times [min]	(*R*)-Limonene 15.2<br>Acetophenone (IS) 16.5<br>(1*S*,5*R*)-Carveol 21.3<br>(1*S*,5*S*)-Carveol 21.6

Table S14: GC-FID parameters used for chiral analytics of (*R*)-(+)-limonene and products.

**Chiral GC-FID parameters – (*R*)-(+)-limonene detection**	
Instrument	Nexis GC-2030 (Shimadzu Europa GmbH.)
Column	Hydrodex-β-6TBDM (Macherey-Nagel)<br>length: 25 m, inner diameter: 0.25 mm, film thickness: 0.25 µM
Injection volume	1 µL
Injection temp.	230°C
Injection mode	Split
Flow control mode	velocity
Pressure	83.1 kPa
Total flow	106.0 mL / min
Column flow	1.02 mL / min
Linear velocity	30 cm / s
Purge flow	3 mL / min
Split ratio	100
Oven temp. program	5 min at 100°C, 15°C min^{-1} to 230°C, 5 min at 230°C
FID temperature	250°C
Compound retention times [min]	(*R*)-Limonene 6.9<br>Acetophenone (IS) 8.7<br>(1*S*,5*R*)-Carveol 10.9<br>(1*R*,5S)-Carveol 11.2<br>(1*S*,5*S*)-Carveol 11.3<br>(1*R*,5*R*)-Carveol 11.8

Table S15: GC-FID parameters used for analytics of indene and the chiral products.

**Chiral GC-FID parameters – indene detection**	
Instrument	Nexis GC-2030 (Shimadzu Europa GmbH.)
Column	Hydrodex-β-6TBDM (Macherey-Nagel)<br>length: 25 m, inner diameter: 0.25 mm, film thickness: 0.25 µM
Injection volume	1 µL
Injection temp.	230°C
Injection mode	Split
Flow control mode	velocity
Pressure	70.1 kPa
Total flow	85.8 mL / min
Column flow	0.82 mL / min
Linear velocity	25,4 cm / s
Purge flow	3 mL / min
Split ratio	100
Oven temp. program	5 min at 100°C, 15°C min^{-1} to 180°C hold 3 min, 15°C min^{-1} to 200°C hold 5 min, 15°C min^{-1} to 230°C hold 5 min
FID temperature	250°C
Compound retention times [min]	Indene 8.1<br>Acetophenone (IS) 9.1<br>(*R*)-1-Indenol 14.5<br>(*S*)-1-Indenol 14.7<br>(1*R*,2*S*)-Indandiol 18.0<br>(1S,2*R*)-Indandiol 18.2<br>(1*R*,2*R*)-Indandiol 19.2<br>(1*S*,2*S*)-Indandiol 19.4

Table S16: GC-FID parameters used for analytics of toluene.

**Chiral GC-FID parameters – toluene detection**	
Instrument	Nexis GC-2030 (Shimadzu Europa GmbH.)
Column	Hydrodex-β-6TBDM (Macherey-Nagel)<br>length: 25 m, inner diameter: 0.25 mm, film thickness: 0.25 μM
Injection volume	1 μL
Injection temp.	230°C
Injection mode	Split
Flow control mode	velocity
Pressure	76.1 kPa
Total flow	115.1 mL / min
Column flow	1.11 mL / min
Linear velocity	30 cm / s
Purge flow	3 mL / min
Split ratio	100
Oven temp. program	5 min at 60°C, 10°C min^{-1} to 230°C, 5 min at 230°C
FID temperature	250°C
Compound retention times [min]	Toluene 5.7<br>Acetophenone (IS) 13.7<br>Benzyl alcohol 15.5

GC-MS

GC-MS analyses were performed on a Shimadzu GCMS-QP2010 SE using a Zebron ZB-5MSi capillary column. Helium was used as carrier gas. Mass spectra were determined in scan mode at a range from 50 to 500 m/z. Additional parameters can be seen in Tables S17 and S18.

Table S17: GC-MS parameters used for qualitative analyses.

**GC-MS parameters – (*R*)-(+)-limonene detection**	
Instrument	GCMS-QP2010 SE (Shimadzu Europa GmbH.)
Column	Zebron ZB-5MSi (Phenomenex Inc.)<br>length: 30 m, inner diameter: 0.25 mm, film thickness: 0.25 μM
Injection volume	1 μL
Injection temp.	250°C
Injection mode	Split
Flow control mode	linear velocity
Pressure	83.8 kPa
Total flow	14.4 mL / min
Column flow	1.13 mL / min
Linear velocity	39.5 cm / s
Purge flow	3 mL / min
Split ratio	9.1
Oven temp. program	4 min at 100°C, 20°C min^{-1} to 340°C, 4 min at 340°C
Compound retention times [min]	(*R*)-Limonene 7.15<br>Acetophenone (IS) 7.6<br>Carveol 9.05
**MS parameters**	
Ion source temp.	250°C
Interface temp.	320°C
Mode:	Scan
Scan range	50 – 500 m / z

Table S18: GC-MS parameters used for qualitative analyses.

**GC-MS parameters – Indene detection**	
Instrument	GCMS-QP2010 SE (Shimadzu Europa GmbH.)
Column	Zebron ZB-5MSi (Phenomenex Inc.)<br>length: 30 m, inner diameter: 0.25 mm, film thickness: 0.25 µM
Injection volume	1 µL
Injection temp.	250°C
Injection mode	Split
Flow control mode	linear velocity
Pressure	70.6 kPa
Total flow	14.8 mL / min
Column flow	1.17 mL / min
Linear velocity	39.5 cm / s
Purge flow	3 mL / min
Split ratio	9.1
Oven temp. program	4 min at 60°C, 10°C min^{-1} to 340°C, 4 min at 340°C
Compound retention times [min]	Indene 8.6<br>Acetophenone 9.0<br>*rac*-Indenol 11.55<br>*cis*-1,2-Indanediol 14.5
**MS parameters**	
Ion source temp.	250°C
Interface temp.	320°C
Mode:	Scan
Scan range	50 – 500 m / z

CALIBRATION

A calibration curve for quantitative analysis using GC-FID was performed. Calibrations were only created for (*R*)-(+)-limonene, (-)-carveol (mixture of isomers) and indene since some of the products were commercially not available. Each calibration consisted of a mixture of the respective compounds in DCM containing 2 mM acetophenone as internal standard. The calibration curve was created for a concentration range from 0.1 to 10 mM of the respective analyte. All calculated peak areas have been normalized to the peak area of the internal standard acetophenone. The quantification of product formation was done by GC-FID ((*R*)-limonenene to carveol) or GC-MS (indene to 1-indenol and *cis*-1,2-indandiol).

Calibration curve for (R)-limonene

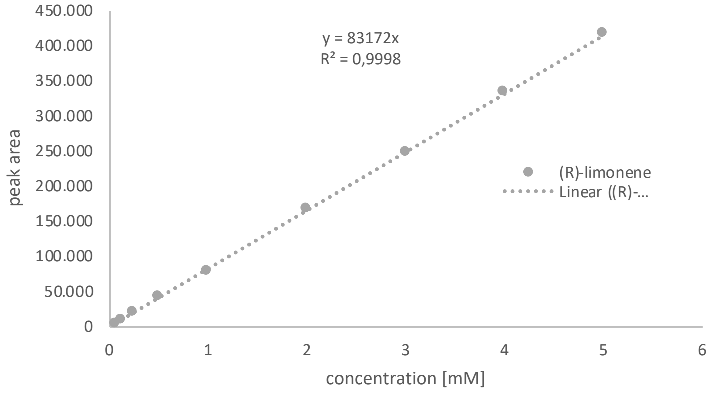

Calibration curve for (5R,5S)-carveol

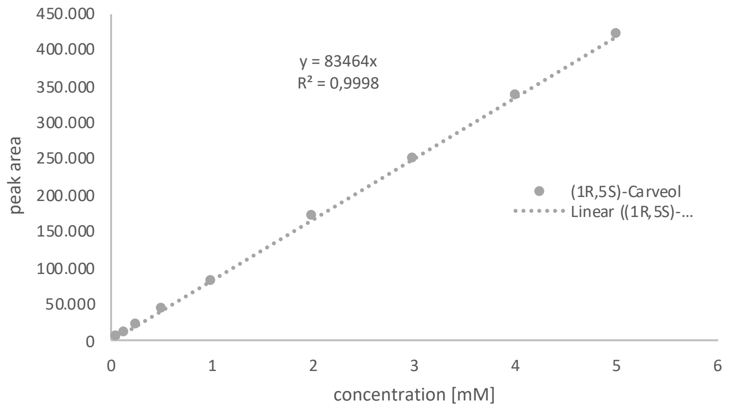

Calibration curve for (1S,5S)-carveol

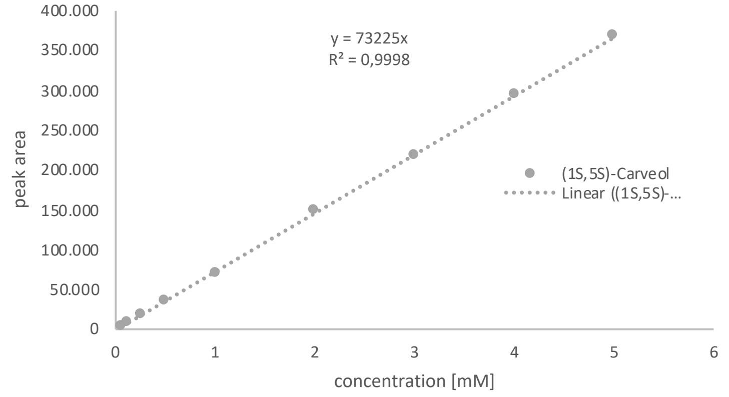

Calibration curve for toluene

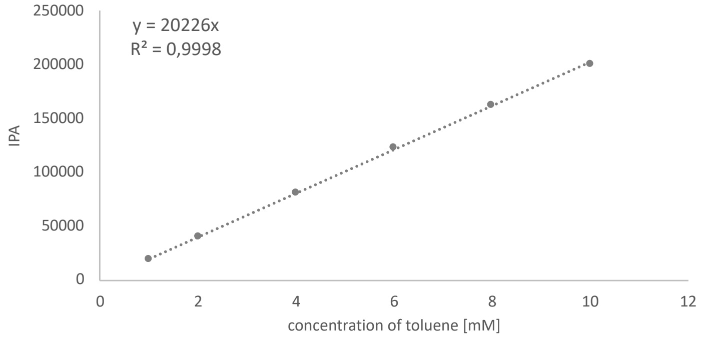

Calibration curve for benzyl alcohol

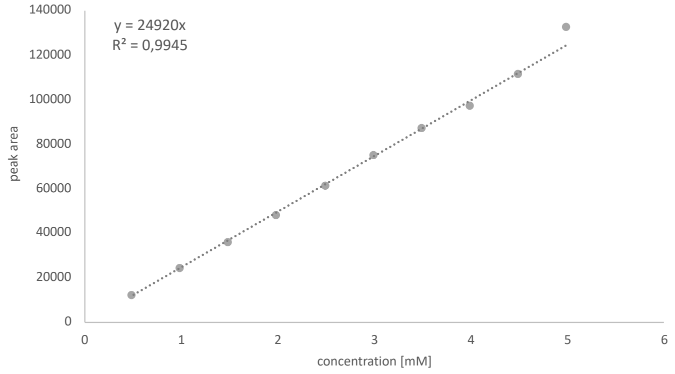

Calibration curve for indene

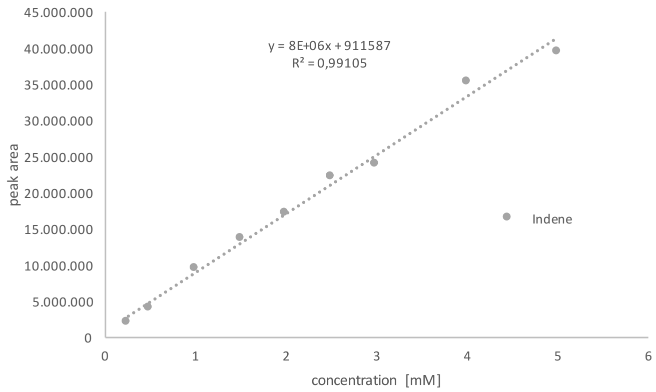

Calibration curve for cis-1,2-indanediol

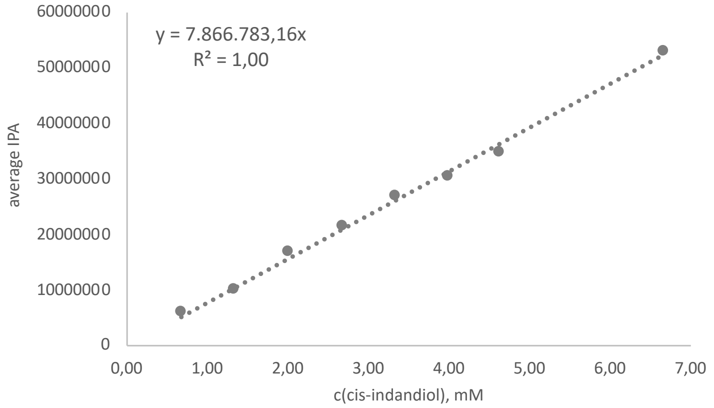

Calibration curve for trans-1,2-indanediol

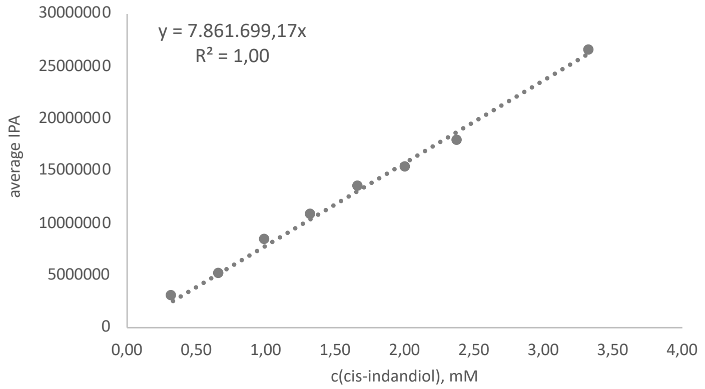

Calibration for 1-indenol

During the synthesis of 1-indenol **3a**, explosive hydroperoxide intermediates (indene-1-peroxide) are formed and hence, relative response factor (RF) values were used to determine concentrations for 1-indenol **3a** in reference to a standard (1-indanone). RF values were calculated from the effective carbon numbers (ECN) of the respective compounds (ECN_x) in relation to the ECN of the standard (ECN_{STD}) using equation (1).[4][5]

$$RF = \frac{ECN_X}{ECN_{STD}} \qquad (1)$$

The concentrations of the analytes were determined by external calibration using standards and normalized with the calculated RF values.

Table S19: Calculated ECN and RF values for quantification of product formation in GC analyses.

Compound	ECN	RF
1-indenol **2a**	9.65	0.93
1-indanone	9	1

Calibration curve for 1-indanone

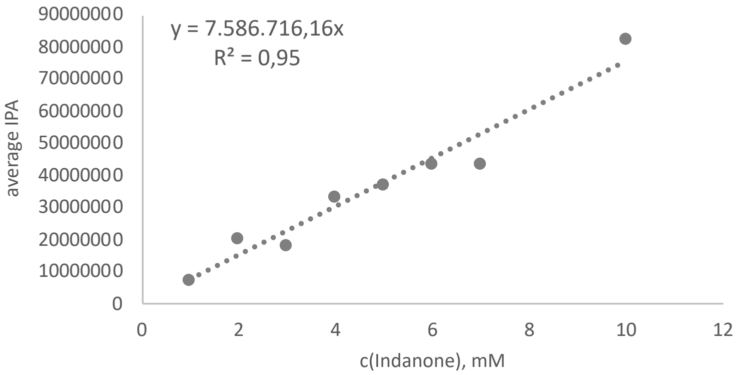

EXAMPLE CHROMATOGRAMS GC-FID AND GC-MS

Carveol

(*R*)-(+)-limonene (Sigma Aldrich) was available with an *ee* of 99%. (1*R*,5*R*)-(-)-carveol and (1*S*,5*R*)-(-)-carveol that were formed in traces as a result of (*S*)-(-)-limonene hydroxylation were not considered for determination of *de* values.

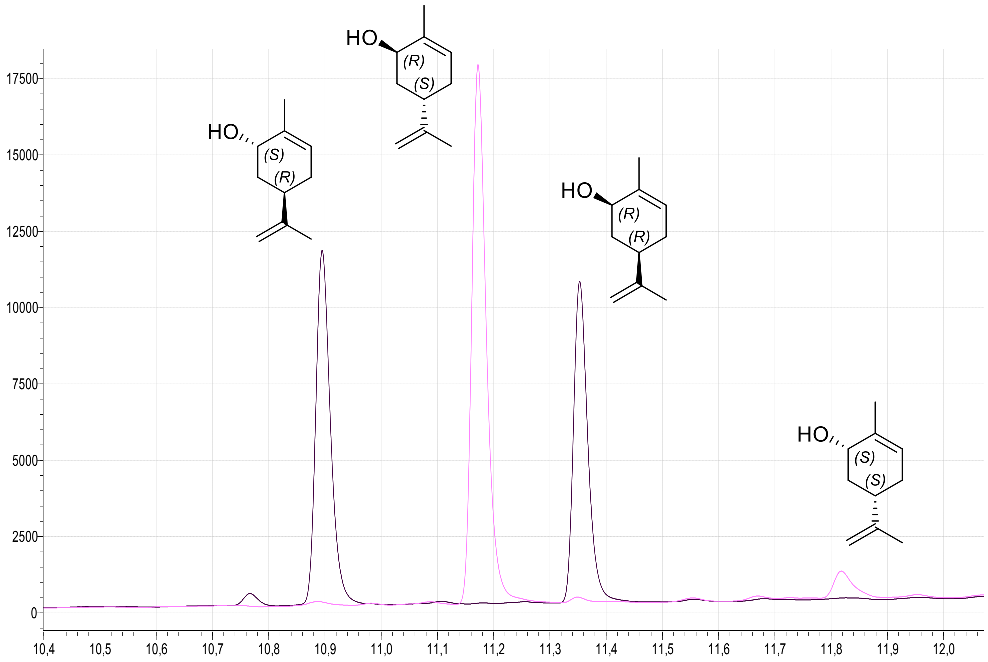

Figure S27: Chiral GC-FID analysis of carveol isomers of the commercial standards or from RO-catalyzed conversion of (*R*)-limonene. Black: racemic mixture of carveol isomers (Sigma Aldrich), (1*R*,5*S*)-carveol (pink), (1*R*,5*R*)-carveol from biotransformations using CDO M232A and (*R*)-limonene.

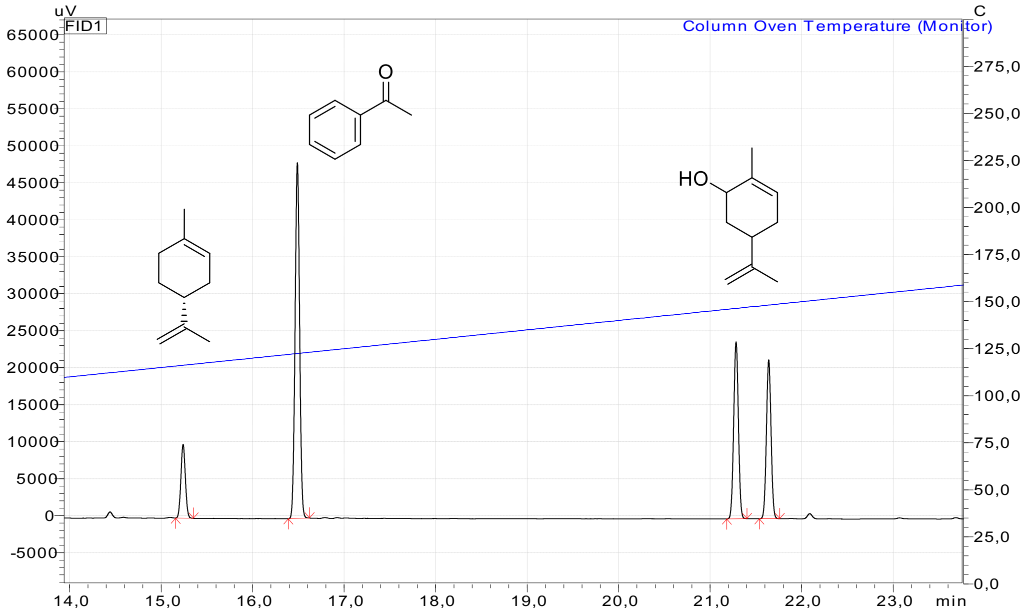

Figure S28: Non-chiral GC-FID analysis of a mixture of (*R*)-limonene, the standard acetophenone and the mixture of commercial carveol isomers.

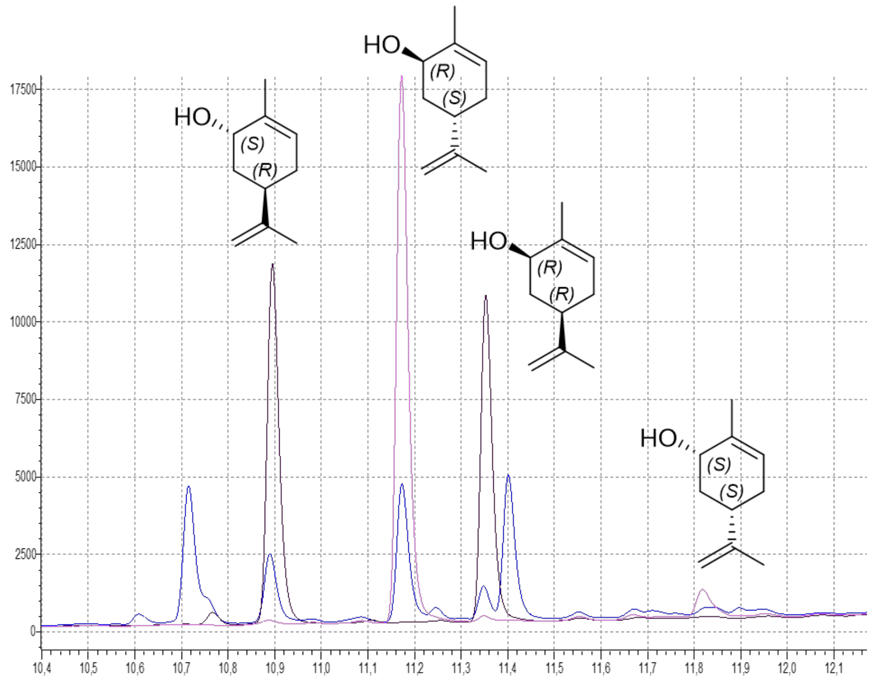

Figure S29: Chiral GC-FID analysis of carveol isomers of the commercial standards (black), from RO-catalyzed conversion of (*R*)-limonene in the dark (pink) and during light-driven biotransformations (blue). Black: racemic mixture of carveol isomers (Sigma Aldrich), (1*R*,5*S*)-carveol obtained from CDO M232A during dark reaction (pink) from (*R*)-limonene, (1*S*,5*R*)-, (1*R*,5*S*)- and (1*R*,5*R*)-carveol from light-driven biotransformations using CDO M232A and (*R*)-limonene (blue).

Indene

Indene was commercially available at Sigma Aldrich. Conversions have been calculated by using:

conversion = $[product]_{final} \times ([product]_{final} + [substrate]_{final})^{-1}$.

Enantiomeric and diastereomeric excess, respectively, for *cis*- and *trans*-indandiol has been determined based on comparison with data known from literature for the NDO- and CDO-catalyzed conversion of indene with the peaks obtained from the commercially available racemic mixture.

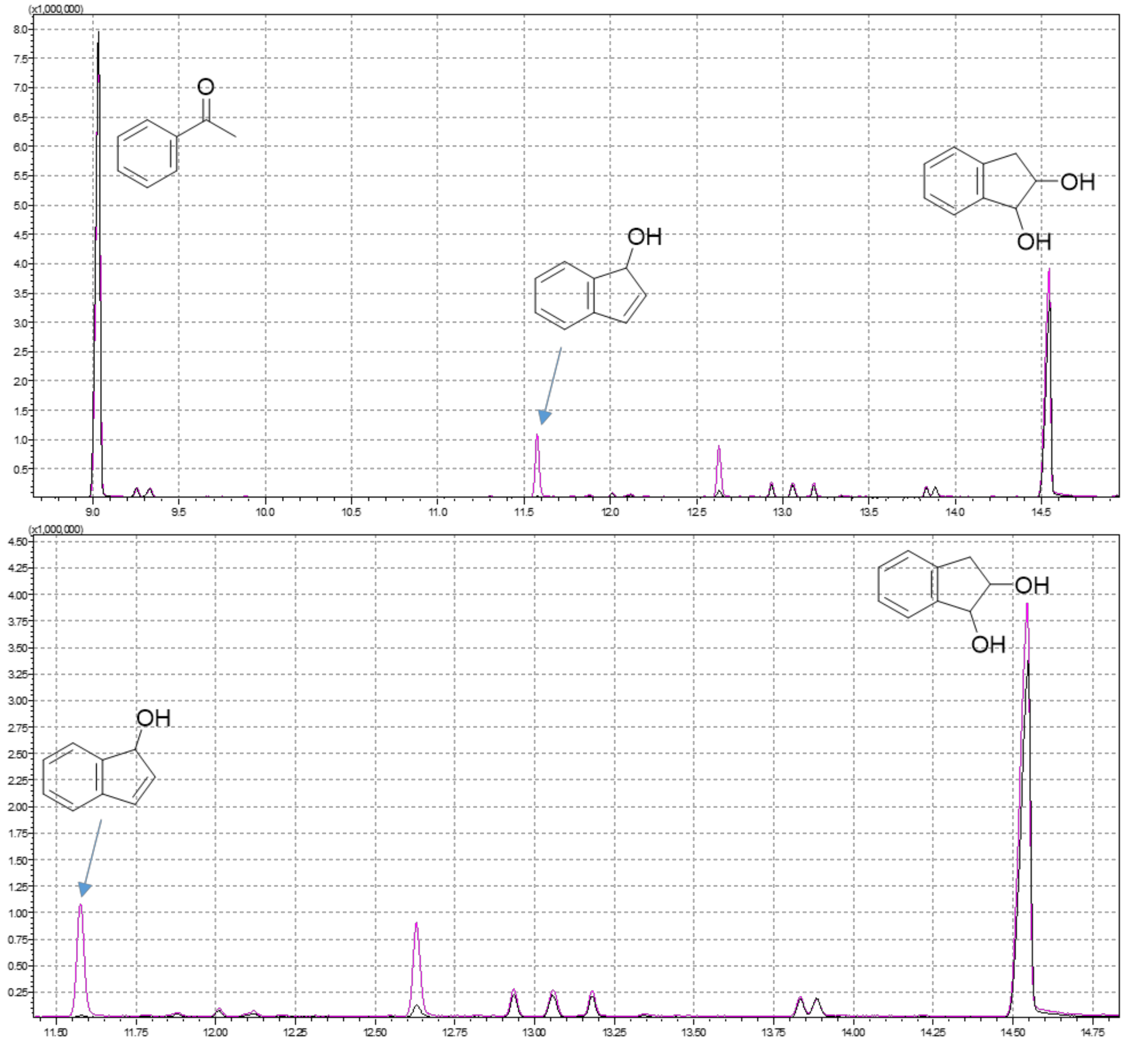

Figure S30: Product distribution of NDO WT (black) and NDO H295A (pink) catalyzed conversion of indene to 1-indenol and *cis*-1,2-indandiol during dark biotransformations.

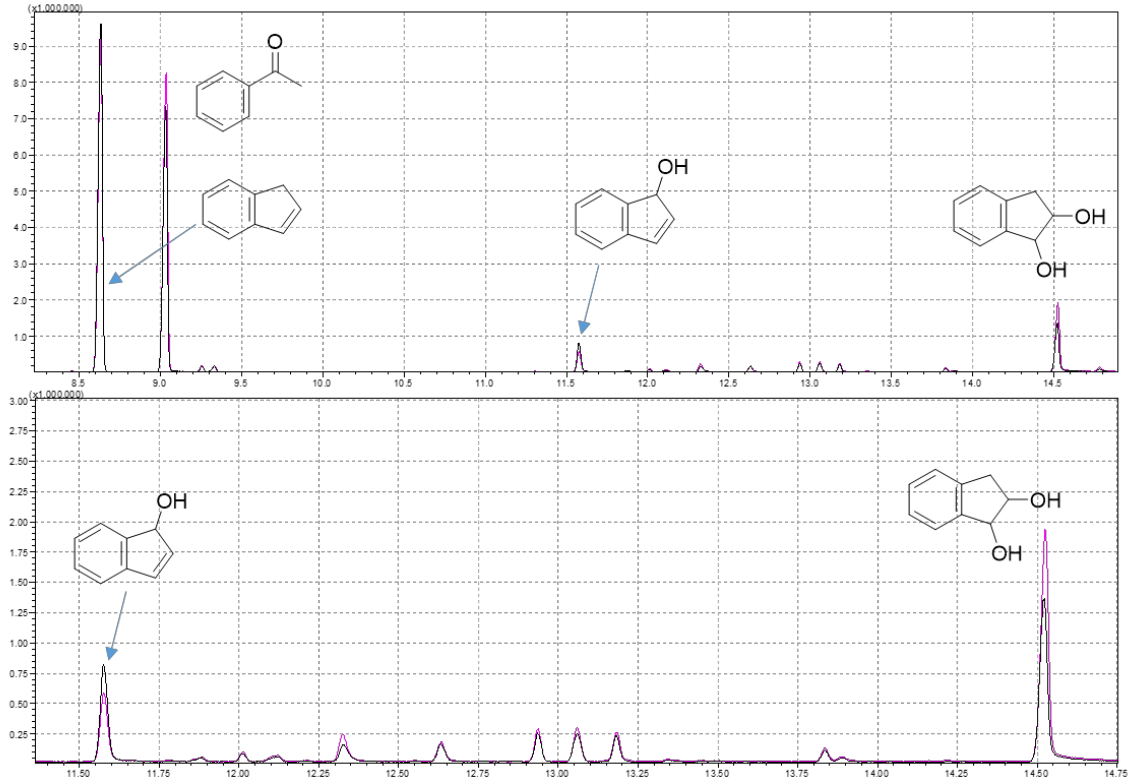

Figure S31: Product distribution of NDO WT (black) and NDO H295A (pink) catalyzed conversion of indene to 1-indenol and *cis*-1,2-indandiol during light-driven biotransformations.

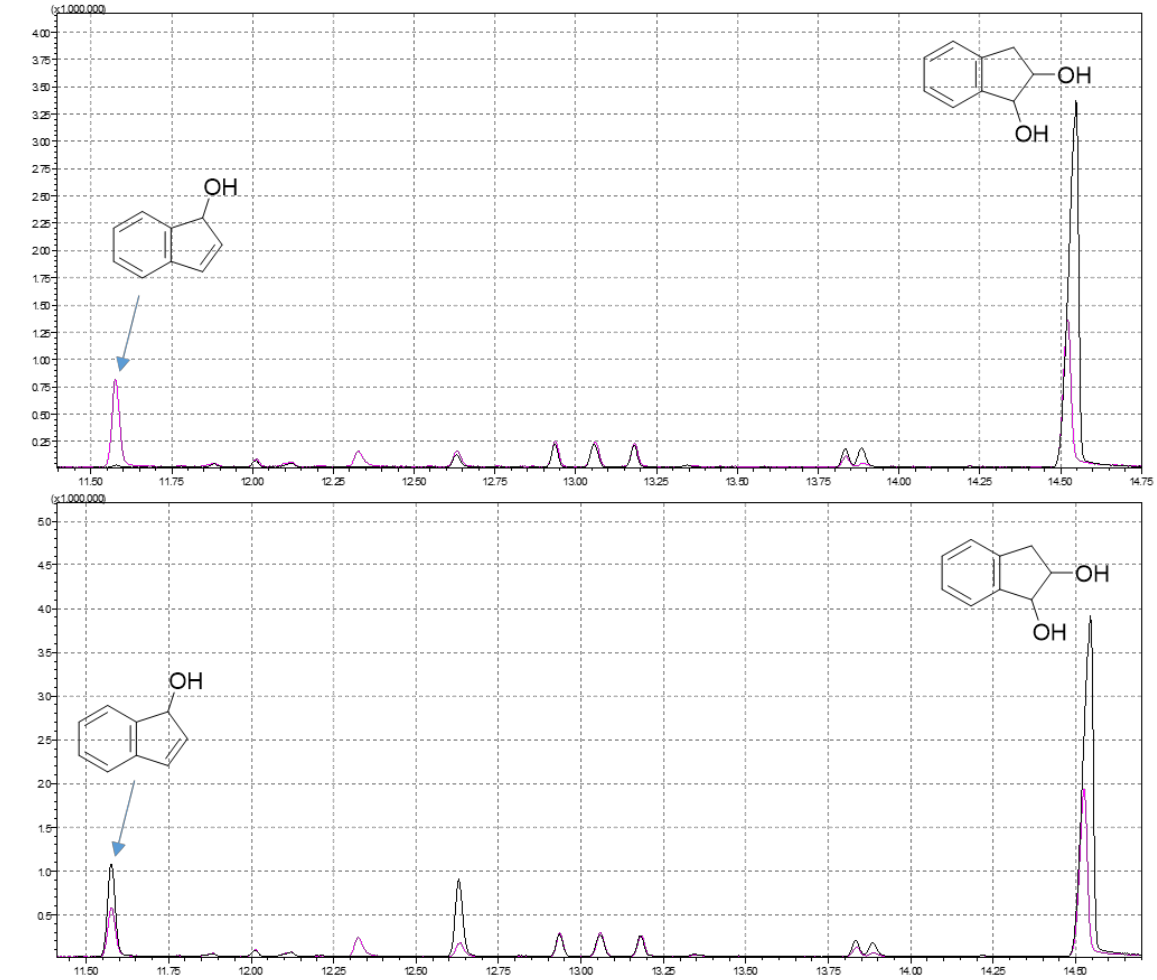

Figure S32: Product distribution of NDO WT (top) and NDO H295A (bottom) catalyzed conversion of indene to 1-indenol and *cis*-1,2-indandiol during dark- (black) and light-driven (pink) biotransformations.

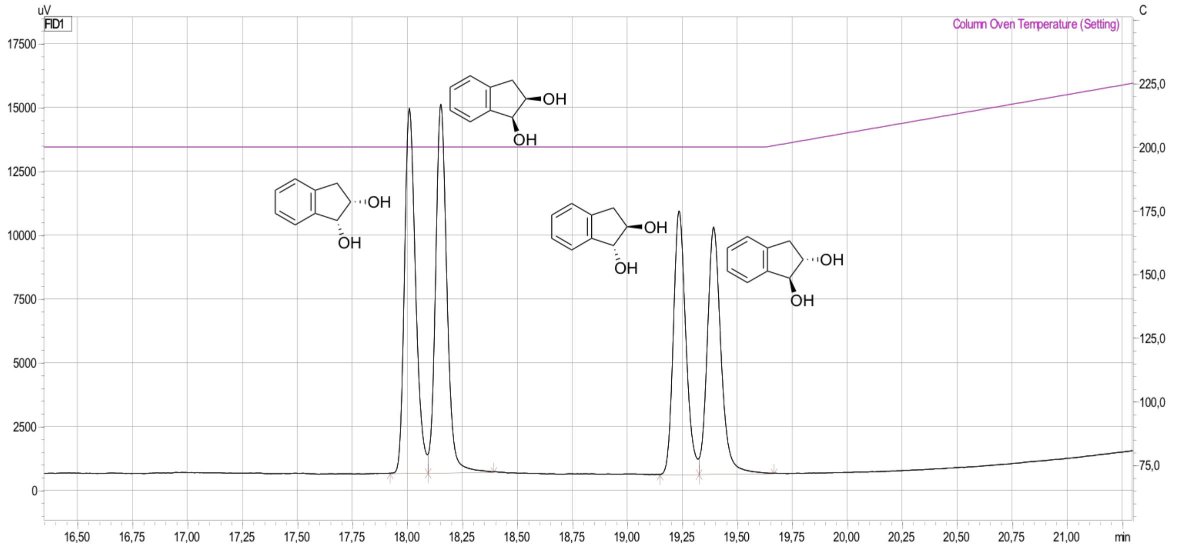

Figure S33: Chiral GC-FID analysis of *cis*- and *trans*-indandiol isomers of the commercial racemic mixture of isomers (Sigma Aldrich).

Toluene

Toluene and benzyl alcohol were commercially available at Sigma Aldrich. Concentrations have been calculated with the help of calibration curves for both, toluene and benzyl alcohol. Conversions have been calculated by using:

conversion = $[product]_{final} \times ([product]_{final} + [substrate]_{final})^{-1}$.

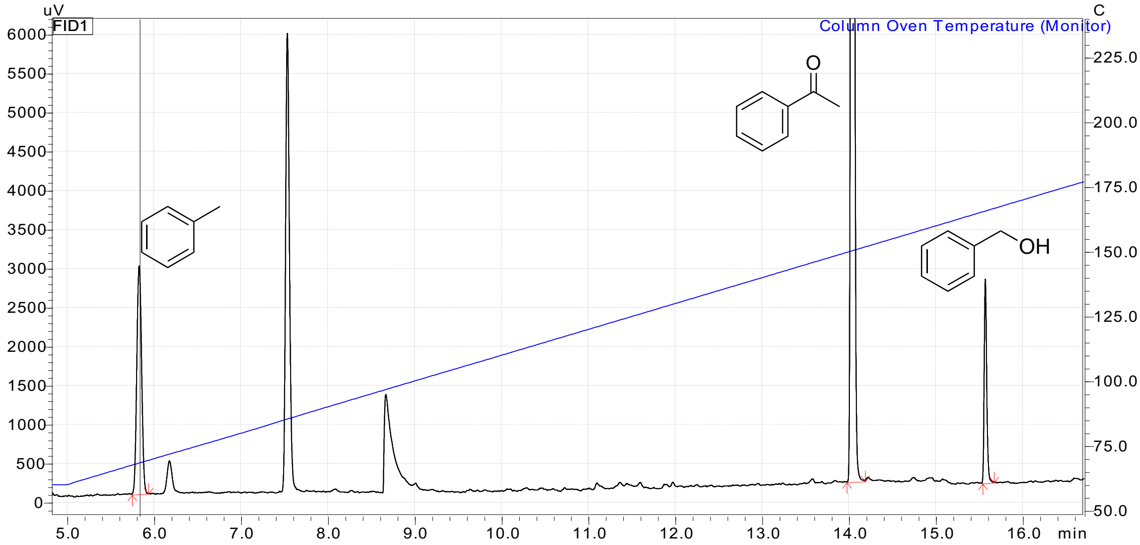

Figure S34: GC-FID analysis of NDO H295A catalyzed conversion of toluene to benzyl alcohol during light-driven biotransformations after 24 hours. The peak at 7.4 min corresponds to an impurity from the solvent ethyl acetate, whereas the peak at 8.6 min corresponds to DMSO.

REFERENCES

[1] R. E. Parales, S. M. Resnick, C. L. Yu, D. R. Boyd, N. D. Sharma, D. T. Gibson, *J. Bacteriol.* **2000**, *182*, 5495–5504.

[2] X. S. Dong, S. Fushinobu, E. Fukuda, T. Terada, S. Nakamura, K. Shimizu, H. Nojiri, T. Omori, H. Shoun, T. Wakagi, *J. Bacteriol.* **2005**, *187*, 2483–2490.

[3] C. Gally, B. M. Nestl, B. Hauer, *Angew. Chem. Int. Ed.* **2015**, *54*, 12952–12956.

[4] J. T. Scanlon, *J. Chromatogr. Sci.* **1985**, *23*, 333–340.

[5] D. E. Willis, J. T. Scanlon, *J. Chromatogr. Sci.* **1985**, *23*, 333–340.

[6] C. G. Hatchard, C. A. A. Parker, *Proc. R. Soc. A Math. Phys. Eng. Sci.* **1956**, *235*, 518–536.
